# Therapeutic potential of alpha-tocopherol in reducing oxidative stress and inflammatory damage after experimental traumatic brain injury and pentylenetetrazol-induced seizures

**DOI:** 10.1007/s00210-026-05442-2

**Published:** 2026-05-19

**Authors:** Cumaali Demirtas, Hava Yildirim, Huseyin Demir, Sezin Kiroglu, Kubra Sevgin, Hakan Beyaztas, Eray Metin Guler, Gulam Hekimoglu, Ugur Aykin, Ender Mehmet Coskunpinar, Mehmet Yildirim

**Affiliations:** 1 Hamidiye Institute of Health Sciences, University of Health Sciences, Istanbul, Turkey; 2 Department of Medical Biology, Hamidiye Institute of Health Sciences, University of Health Sciences, Istanbul, Turkey; 3 Department of Neurosurgery, Istanbul Health and Technology University, Istanbul, Turkey; 4 Department of Physiology, Hamidiye Faculty of Medicine, University of Health Sciences, Istanbul, Turkey; 5 Department of Histology and Embryology, Hamidiye International Faculty of Medicine, University of Health Sciences, Istanbul, Turkey; 6 Department of Medical Biochemistry, Hamidiye Faculty of Medicine, University of Health Sciences, Istanbul, Turkey; 7 Department of Medical Biochemistry, Hamidiye Institute of Health Sciences, University of Health Sciences, Istanbul, Turkey; 8 Department of Medical Biochemistry, Haydarpasa Numune Health Application and Research Center, University of Health Sciences, Istanbul, Turkey; 9 Department of Medical Biology, Hamidiye Faculty of Medicine, University of Health Sciences, Istanbul, Turkey

**Keywords:** Traumatic brain injury, Pentylenetetrazol-induced seizures, Alpha-tocopherol, Oxidative stress, MiRNA

## Abstract

The effects of alpha-tocopherol on seizure parameters, locomotor-cognitive functions, inflammatory response, oxidative stress response, histopathological changes, immunohistochemical parameters, and miRNA fold changes were investigated in rats with traumatic brain injury (TBI) and pentylenetetrazol (PTZ)-induced seizures. Sprague–Dawley male rats were randomly divided into three groups: Control (*n* = 8), TBI + PTZ (*n* = 10), and TBI + PTZ + tocopherol (*n* = 10). After inducing TBI in animals using the weight-drop method, increased post-injury seizure susceptibility was achieved by administering subconvulsive doses of PTZ. Saline was administered intraperitoneally to the control and TBI + PTZ groups for 6 days, while 500 mg/kg alpha-tocopherol was administered intraperitoneally to the TBI + PTZ + tocopherol group. Seizure intensity, seizure frequency, and total seizure duration were significantly reduced in the TBI + PTZ + tocopherol group compared to the TBI + PTZ group (*p* < 0.05). No significant adverse effects related to TBI and PTZ were observed in the animals’ locomotor activity, anxiety-like behaviors, or learning and memory test outcomes. In the TBI + PTZ + tocopherol group, significant reductions were observed in inflammatory cytokine response, oxidative stress, and SUR1-TRPM4 channel activity compared to the TBI + PTZ group (*p* < 0.001). While degenerative and apoptotic neurons and the number of 8-OHdG-positive cells in the CA1 and dentate gyrus regions were limited in the TBI + PTZ + tocopherol group, downregulated miR-324-5p increased (*p* < 0.05). Alpha-tocopherol reduced the severity and duration of seizures, reduced oxidative stress and inflammation, and stabilized the thiol-disulfide balance. It also reduced degenerative cell structures and DNA damage in the cortex, hippocampus, and dentate gyrus. In conclusion, the findings of this study suggest that alpha-tocopherol is a potential neuroprotective agent that modulates early epileptogenic network instability in TBI and seizure susceptibility through multiple pathways, including oxidative stress, inflammation, and ion channel regulation.

## Introduction

Traumatic brain injury (TBI), defined as pathological changes in the brain caused by external physical forces, is a health problem that is increasing worldwide due to motor vehicle use, sports activities, and warfare, causing injuries that result in death and disability, especially among adults (Dang and Wang 2023). Despite advances in accident prevention and early post-accident resuscitation techniques, it remains a significant health problem in terms of long-term neurological morbidity (Khayatan et al. 2024). Studies of TBI patients admitted to trauma units have examined cases ranging from mild to severe, and a trend toward more severe cases has been identified (Christensen et al. 2009). TBI has been categorized into mild, moderate, and severe categories, and the severity of the TBI has been associated with the risk of developing seizures, with severe cases demonstrating a significantly higher cumulative risk over 20 years (Annegers et al. 1998). Posttraumatic epilepsy (PTE), characterized by recurrent, unprovoked seizures and one of the most common and disabling consequences of TBI, is seen in patients with TBI of varying severity and occurs more frequently after severe trauma, leading to significant negative impacts on the patient’s quality of life and safety (Pingue et al. 2021). According to the available literature, there is no effective treatment for TBI approved by any relevant authorities or institutions (Taylor 2017). Due to the complex and heterogeneous pathophysiological process of TBI, traditional methods for diagnosing and treating TBI are inadequate, and therefore, alternative methods are needed to develop an effective treatment for TBI (Khayatan et al. 2024).

Primary (mechanical) damage occurring at the time of TBI injury is responsible for focal anatomic lesions such as lacerations, contusions, intracranial hemorrhage, and widespread axonal injury, whereas secondary (non-mechanical, delayed) damage beginning at the time of injury does not cause clinical changes for hours or days (Cornelius et al. 2013). Pathologies associated with secondary damage include metabolic changes, neuroinflammation, axonal injury, vascular abnormalities, and neuronal and glial cell death (Donkin and Vink 2010). Excitotoxicity is a pathological condition associated with neuronal death and oxidative stress that occurs secondary to acute brain injury (Clausen et al. 2012). Oxidative stress results from an imbalance between the biochemical processes that lead to the production of reactive oxygen species (ROS) and the processes involving the antioxidant cellular defense system responsible for scavenging ROS (Frati et al. 2017). Oxidative stress has been suggested to lead to brain edema due to mitochondrial dysfunction, blood–brain barrier (BBB) disruption, sensory-motor dysfunction, and secondary neuronal damage (Fesharaki-Zadeh 2022). There is substantial evidence that oxidative stress is both an initiator and perpetuator of the pathological processes related to secondary damage following TBI, and acute antioxidant supplementation has been reported to be successfully used to reduce brain damage even in the subacute phases of TBI (Fernández-Gajardo et al. 2014).

Vitamin E is a group of chemicals composed of eight different compounds: α-, β-, γ-, and δ-tocopherols and tocotrienols, which function as lipophilic radical-scavenging antioxidants (Niki 2021). Alpha-tocopherol transfer protein (α-TTP) is the most biologically active form of vitamin E, specifically recognizing alpha-tocopherol and binding it with high affinity to other vitamin E components to promote its secretion into circulating lipoproteins (Noguchi and Niki 2024). Alpha-tocopherol is an important component of the antioxidant defense system due to its physical–chemical properties, the presence of a chromanol ring, lipid solubility, its ability to act as a nonspecific chain-breaking antioxidant, and its cooperation with a network of endogenous and exogenous antioxidants (Dobrovolny et al. 2018). Alpha-tocopherol is a typical peroxyl radical scavenger, inhibiting the oxidative chain by generating the highly stable α-tocopheroxyl radical after its reaction with the peroxyl radical (ROO-) and protecting polyunsaturated fatty acids within membrane phospholipids, particularly arachidonic acid and docosahexaenoic acid (Burton et al. 1982). Due to these properties, alpha-tocopherol has been reported to protect cell membrane integrity by reducing lipid peroxidation induced by lipopolysaccharide, microglia, and interleukin-6 (IL-6), exhibit neuroprotective effects, preserve synaptic plasticity, and lead to a significant increase in the levels of neuroregenerative and anti-inflammatory components (Ambrogini et al. 2014; da Cunha et al. 2023; Demirtas et al. 2025a).

There are no studies in the literature evaluating the efficacy of alpha-tocopherol, which possesses potent antioxidant capacity, on TBI and PTZ-induced seizures at the behavioral, biochemical, molecular, and histopathological levels. The presented study investigated the effects of alpha-tocopherol, administered immediately after trauma and throughout the study, in an experimental post-injury seizure susceptibility model induced by subconvulsant pentylenetetrazol (PTZ) injection after TBI induced by weight drop. Physiological examinations included behavioral seizure scoring, locomotor activity, spatial memory, and anxiety tests. Biochemical studies measured oxidative stress parameters. Molecular studies identified microRNA (miRNA) levels associated with trauma and epilepsy. Histopathological examinations examined the level of neuronal damage in brain tissue. This study is unique in that it is the first comprehensive, multidisciplinary study to examine the efficacy of alpha-tocopherol in TBI and PTZ-induced seizures.

## Material and methods

### Animals

A total of 28 male Sprague–Dawley rats, weighing an average of 250 ± 30 g, were used in the experiments. The animals were bred and obtained from the University of Health Sciences Hamidiye Experimental Animal Production and Research Laboratory. The experimental animals were reared in housing conditions with an ambient temperature of 21 ± 2 °C and humidity of 50–60%, maintaining a 12/12-h light–dark cycle. Four animals were housed in each cage, provided with standard pelleted diet and tap water, with access to adequate food and water without any dietary restrictions. All procedures in this study were performed in accordance with Directive 2010/63/EU of the European Parliament and of the Council on the Protection of Laboratory Animals Used in Scientific Research. The experimental procedure was approved by the University of Health Sciences’ Hamidiye Local Animal Experimentation Ethics Committee (Approval No: 25–13).

### Experimental design

The study is based on an experimental post-injury seizure susceptibility model created in rats by provoking them with subconvulsant doses of PTZ after TBI (Efendioglu et al. 2020). Regarding experimental epilepsy models, different PTE models are available in the literature, varying depending on the severity and type of trauma, as well as the observation period for PTE development (Pitkänen et al. 2009). While studies lasting up to 6–12 months have been designed to observe the spontaneous development of seizures due to trauma (Pitkänen et al. 2009), it appears that sufficient standardization has not been achieved in the application of these models. However, in a previous study conducted in our laboratory, subconvulsant doses of PTZ were administered to standardize the post-injury seizure susceptibility model and the contribution of TBI to seizure development was clearly demonstrated (Efendioglu et al. 2020). In this study, instead of waiting a long time for seizures to develop spontaneously after TBI, the development of trauma-related seizures was facilitated with subconvulsant PTZ.

**Fig. 1:**
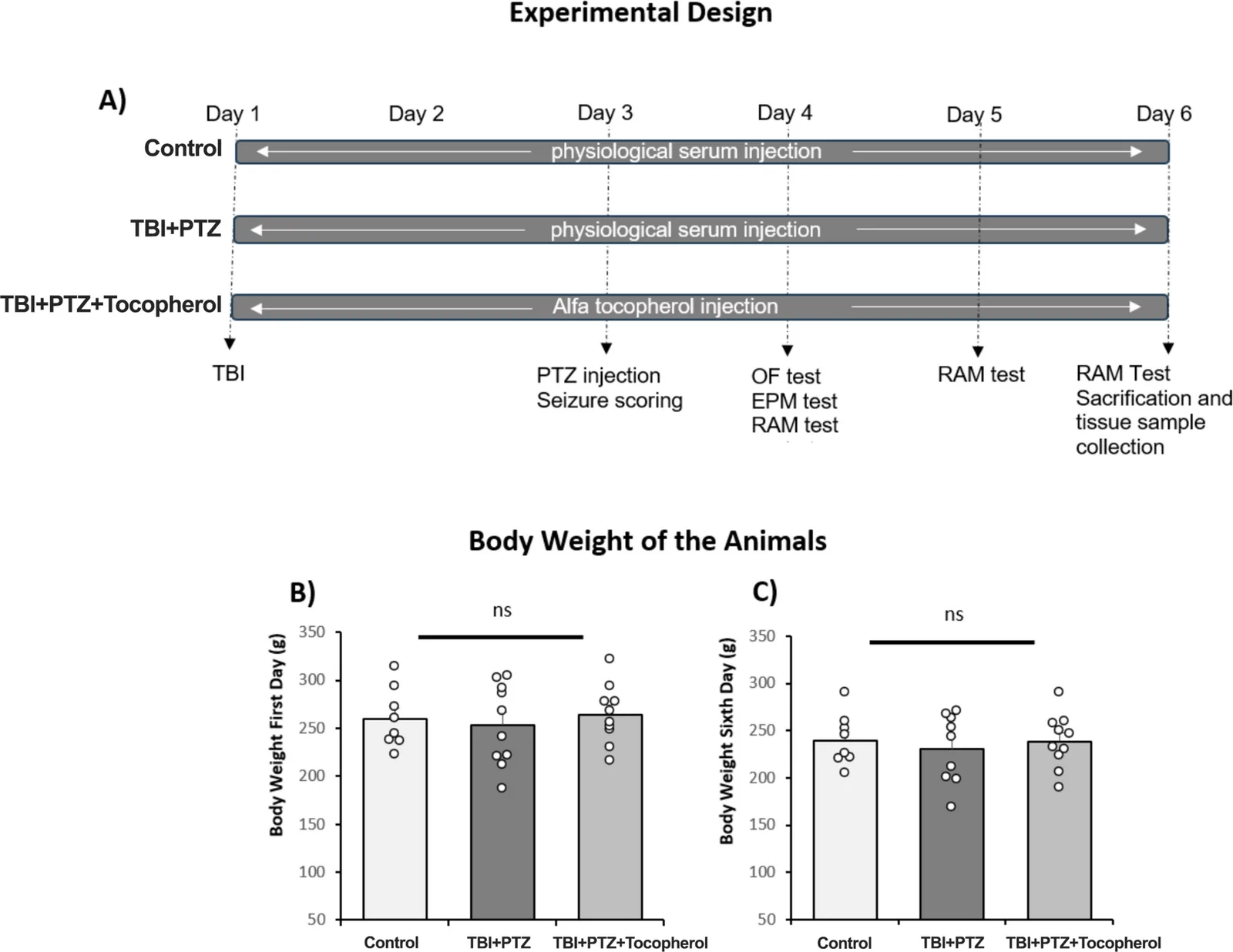
Experimental design and body weight of the animals. **A** Experimental timeline, study groups, and procedures performed. **B** Initial body weights of rats at the beginning of the experiment. **C** Final body weights of rats at the end of the experiment

Beginning immediately after the trauma model was established, the animals received daily intramuscular alpha-tocopherol at a dose of 500 mg/kg throughout the study period. The alpha-tocopherol dose was determined based on literature studies (Kozan et al. 2006, 2007; Ayyildiz et al. 2007). Two hours before subconvulsant PTZ administration, the animals received a third dose of alpha-tocopherol (Fig. 1A).

**Fig. 2:**
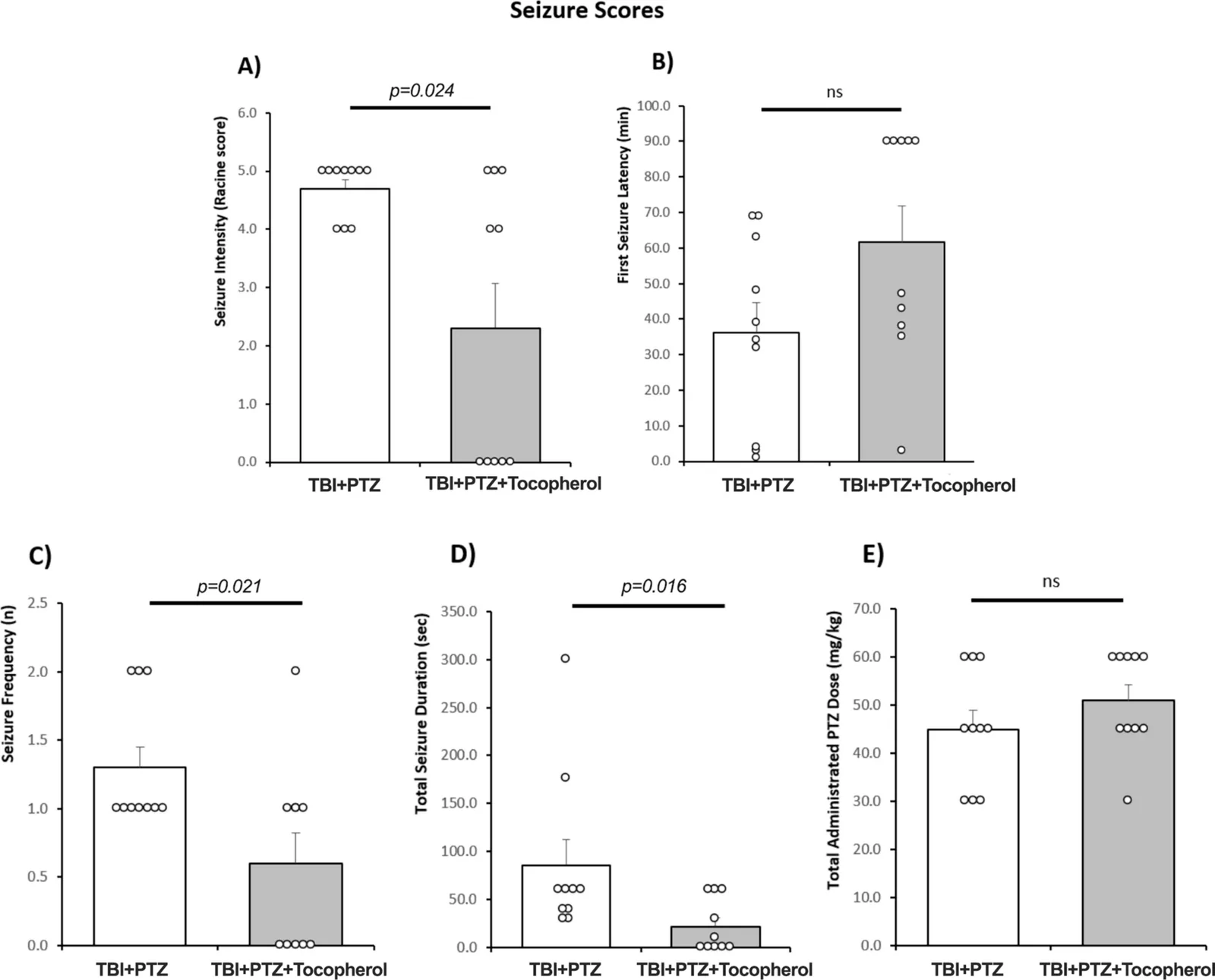
Seizure scores. **A** Seizure intensity (Racine stage 4 and 5 seizures) was reduced in the TBI + PTZ + Tocopherol group compared to the TBI + PTZ group. **B** The latency to the first seizure was longer in the TBI + PTZ + Tocopherol group, although the difference was not statistically significant compared to the TBI + PTZ group. **C** Seizure frequency was significantly decreased in the TBI + PTZ + Tocopherol group. **D** Total seizure duration was significantly reduced in the TBI + PTZ + Tocopherol group compared to the TBI + PTZ group. **E** The total PTZ dose required to induce seizures was higher in the TBI + PTZ + Tocopherol group, but the difference was not statistically significant compared to the TBI + PTZ group. Note:TBI + PTZ group (*n* = 10); TBI + PTZ + Tocopherol group (*n* = 10). Data are given with mean ± SEM. Statistical analyses were performed after the Kruskal-Walli’s analysis of variance, using the Mann–Whitney* U* post hoc test for pairwise group comparisons: *p*-value < 0.05 was considered statistically significant. Bonferroni correction was not applied because only two groups were compared

On the first day of the study (day 1), the experimental animals were anesthetized with 3% sevoflurane and then subjected to head trauma using weight drop. A painkiller (paracetamol) with no anti-inflammatory effect was added to the drinking water for 24 h after the head trauma. Forty-eight hours after the head trauma (day 3), seizures were induced by intraperitoneal (i.p.) injection of 30 mg/kg subconvulsant PTZ. If the initial dose was insufficient to induce a seizure at Racine 4 or 5, a maximum of two more 15 mg/kg subconvulsant PTZ injections were administered at 30-min intervals. A total of 90 min of observation was conducted, and seizure parameters were scored according to the Racine scale. To determine the effects of TBI and PTZ-induced seizure-related neuronal damage and the effects of alpha-tocopherol, an open-field test was performed to examine locomotor activity 24 h after seizure scoring (day 4), and an elevated plus maze test was performed to determine anxiety level. The radial arm maze test, which examined spatial memory, was performed for 3 days (days 4, 5, and 6). After behavioral tests (day 6), the experimental animals were anesthetized with 3% sevoflurane and sacrificed, and intracardiac blood and brain tissue were collected (Fig. 1A). Researchers were blind to the groups when examining blood and tissue samples taken from the animals.

### Experimental groups

The animals were randomly assigned to three groups, with 8 animals in the control group and 10 animals in TBI + PTZ and TBI + PTZ + Tocopherol groups. Sample size estimation was performed using a one-way ANOVA framework (*α* = 0.05, power = 80%, effect size *f* = 0.40) and 28 animals were included due to ethical restrictions. According to the power analysis, 8 animals were needed per group, but due to concerns about deaths from traumatic brain injury, 2 more animals were added to the groups that received traumatic brain injury; however, since traumatic brain injury was well standardized, the expected losses did not occur.

- *Control group (n* = *8)*: Serum physiologic (2 ml/kg) was administered intraperitoneally for 6 days, the study period, without the use of a trauma model. Following the behavioral tests, blood and brain tissue samples were collected.
- *TBI* + *PTZ group (n* = *10)*: Serum physiologic (2 ml/kg) was administered intraperitoneally for 6 days, the study period, starting immediately after the use of a trauma model. Seizure scoring was performed 48 h after the head injury by injecting 30 + 15 + 15 mg/kg PTZ (2 ml/kg, i.p.). Following the behavioral tests, blood and brain tissue samples were collected.
- *TBI* + *PTZ* + *Tocopherol group (n* = *10)*: Alpha-tocopherol (500 mg/kg) was administered intramuscularly for the 6-day study period, beginning immediately after the trauma model. Seizure scoring was performed 48 h after the head injury by injecting 30 + 15 + 15 mg/kg PTZ (2 ml/kg, i.p.). Following the behavioral tests, blood and brain tissue samples were collected.

### Traumatic brain injury and PTZ-induced seizures

#### Establishment of traumatic brain injury model

Mild head trauma was induced in rats using the experimental trauma model developed by Marmarou and colleagues (Marmarou et al. 1994)using the weight drop method. Animals were anesthetized with 3% sevoflurane and then subjected to head trauma using weight drop. The trauma apparatus consisted of a hollow glass tube attached to a foot stand and extending vertically to the floor. The anesthetized rat was placed prone on a Plexiglas cage covered with aluminum foil. The rat’s head was aligned 1 cm below the glass tube, parallel to the floor along the rostral-caudal axis. Closed head trauma was induced by dropping a 250 g stainless steel weight onto the midline of the head from a height of 100 cm through the glass tube. The steel weight was dropped directly onto a 10 mm diameter and 3 mm thick stainless-steel disk placed on the animal’s scalp, rather than on the scalp. The weight was attached with a rope and scaled to prevent a second impact (Marmarou et al. 1994; Fehily et al. 2019; Dill et al. 2022). A painkiller (paracetamol) with no anti-inflammatory effect was added to the drinking water for 24 h after the head trauma.

#### PTZ-induced seizures

Seizures was induced by administering a subconvulsant dose of PTZ 48 h after TBI. Subconvulsant doses of 30 + 15 + 15 mg/kg of PTZ (2 ml/kg, i.p.) were administered incrementally until a seizure occurred at a Racine 4 or 5 level. If no seizures occurred at a Racine 4/5 level, a second dose of 15 mg/kg of PTZ was administered 30 min after the first 30 mg/kg dose of PTZ. If no seizures occurred again, a third dose of 15 mg/kg of PTZ was administered 30 min later (Efendioglu et al. 2020; Aykin et al. 2025).

#### Behavioral seizure scoring

Behavioral seizure parameters were scored for 90 min after PTZ using the modified Racine scale (Basaran et al. 2025). Seizure severity was assessed according to the modified Racine scale as follows; stage 0: no response; stage 1: ear and facial twitching; stage 2: convulsive fluctuations in body posture; stage 3: myoclonic jerks with rearing; stage 4: tonic–clonic convulsions with return to the lateral position; stage 5: generalized tonic–clonic seizures with loss of postural control (Efendioglu et al. 2020; Aykin et al. 2025). The scoring process was performed by the researchers in real time, and seizure-related parameters were checked on the video recordings afterward. As a result of the scoring, seizure latency, seizure frequency, seizure duration, and seizure severity were determined for use in the analyses related to behavioral seizure parameters. Seizure severity was calculated as the highest seizure severity according to the Racine scale occurring during the total of 90 min after PTZ injection. Seizure latency was determined as the time until the first seizure occurred at a Racine scale score of 4 or 5 within the first 30 min after 30 mg/kg PTZ administration. If seizures did not occur at a Racine scale score of 4 or 5 with the first dose, additional doses (two 15 mg/kg doses) were administered, and the elapsed time was included in the latency calculation. Seizure frequency was calculated as the total number of seizures at a Racine scale score of 4 and 5 occurring during the total of 90 min after PTZ injection. Total seizure duration was determined as the total duration of seizures at levels 4 and 5 on the Racine scale occurring for a total of 90 min after PTZ injection. The total PTZ dose administered for each animal was also calculated (Efendioglu et al. 2020; Aykin et al. 2025).

### Locomotor and cognitive function tests

#### Open field test

The open field test setup consisted of a 100 × 100 cm and 35 cm high, gray, Plexiglas-shaped square area where rats’ locomotor activity could be observed. Experiments were conducted between 9:00 and 15:00 under dim light. Object tracking software (ANY-Maze Video Tracking System, Stoelting Europe, Dublin, Ireland) was used to record and analyze behavioral parameters. Each rat was released from a designated corner of the open field test setup, and their behavior was recorded for 300 s. The total distance travelled and number of rearing were used as a measure of locomotor activity. The number of entries into the central area, the time spent in this area, total immobility time, and the number of defecations were considered indicators of anxiety behaviors (Racine 1972; Demirtas et al. 2025b; Mazi et al. 2025).

#### Radial arm maze test

The radial arm maze test apparatus consists of an eight-arm radial maze used to assess spatial learning and memory processes. The experiment was conducted in three phases: habituation, learning, and testing. During the habituation phase, rats were left in the apparatus for 10 min to allow them to become accustomed. Following this phase, the rats were food-restricted for 24 h. During the learning phase, which took place the following day, food was placed in the last section of only one of the eight arms. The sliding doors of the other arms were closed. The rat was placed in the center of the apparatus, facing away from the relevant test arm. For 10 min, the rat was allowed to freely enter and exit the baited arm and feed. Following this stage, the rats were again food restricted. During the test phase the next day, the rat was placed in the center of the apparatus, facing away from the relevant test arm. The sliding doors of all arms were opened, allowing free access to all arms. After the animal was placed in the apparatus, its latency to find the arm containing the food the day before and the number of incorrect entries into the other arms during this time were recorded for 300 s. Object tracking software (ANY-Maze Video Tracking System, Stoelting Europe, Dublin, Ireland) was used to record and analyze all behavioral parameters except when the animals were placed in and retrieved from the apparatus (Aykin et al. 2025; Demirtas et al. 2025b).

#### Elevated plus maze test

The elevated plus maze test is a method used to measure anxiety levels in experimental animals. This test method is based on the animal's avoidance of open spaces. Anxiety levels are expressed by the animal spending more time in closed arms. The elevated plus maze apparatus is made of gray Plexiglas and consists of four arms, two open and two closed, in a plus shape, 60 cm above the ground. Each of these arms is 10 cm wide and 50 cm long. The wall height of the closed arms is 40 cm. The total length of the open corridor, including the central section where the four arms intersect, is 110 cm. The rat was placed at the junction of the four arms of the maze, facing a predetermined open arm. The behavior of the animal tested in the elevated plus maze was monitored and recorded using object tracking software (ANY-Maze Video Tracking System, Stoelting Europe, Dublin, Ireland). The number of entries into each arm and the time spent in each arm were automatically determined for 300 s using ANY-Maze software. An increase in closed arm activity (duration and/or entries) indicates an increase in anxiety level, while an increase in open arm activity (duration and/or entries) indicates a decrease in anxiety level (Aykin et al. 2025; Mazi et al. 2025).

### Biochemical analyses

#### Collection of serum and tissue samples

Brain tissues obtained from rats were stored at − 80 °C until biochemical analyses. Intracardiac blood samples were collected in biochemistry tubes containing gel and centrifuged at 3000 × *g* for 10 min using a Beckman Coulter Allegra® X-30 centrifuge (IN, USA). The resulting serum samples were aliquoted and stored at − 80 °C until analysis.

#### Tissue homogenization and total protein measurement

Tissue samples were brought to room temperature and homogenized in 1 × phosphate-buffered saline (PBS; 0.1 M, pH 7.4) using a QIAGEN TissueLyser LT instrument (Hilden, Germany). The resulting homogenates were centrifuged at 13,000 rpm for 10 min using a Beckman Coulter Allegra® X-30 centrifuge (USA), and the supernatant was separated for analysis. Total protein concentration was measured using the Coomassie Plus Protein Assay Kit (Thermo Fisher Scientific, MA, USA), and absorbance values were read at 595 nm with a BioTek Synergy™ HTX multimodal reader. Protein concentrations were calculated using a standard curve generated with known standard proteins. Oxidative stress and inflammation parameters measured in tissue samples were normalized to total protein levels to compensate for differences in sample concentration.

#### Total antioxidant level measurement

Total antioxidant level (TAS) levels were measured using a commercial colorimetric assay kit (Rel Assay Diagnostics, RL0017, Türkiye). The method is based on the formation of the hydroxyl radical (•OH) via the Fenton reaction between Fe^2^⁺-o-dianisidine and hydrogen peroxide (H₂O₂). Antioxidants present in the sample prevent the color development caused by these radicals, and this color change is measured spectrophotometrically and expressed as μmol Trolox equivalents/L (μmol Trolox Eqv./L).

#### Total oxidant level measurement

Total oxidant level (TOS) levels were measured using a commercial colorimetric analysis kit (Rel Assay Diagnostics, RL0024, Turkey). The method is based on the oxidants in the sample oxidizing the ferrous ion–o-dianisidine complex to ferric ion. Ferric ions formed in an acidic environment form a colored complex with xylenol orange. The resulting color intensity is directly proportional to the total oxidant concentration and is measured spectrophotometrically and expressed as μmol hydrogen peroxide equivalents/L (μmol H₂O₂ Eqv./L). Oxidative stress index calculation: OSI was calculated using the formula: OSI (AU) = TOS (μmol H₂O₂ Eqv./L)/TAS (mmol Trolox Eqv./L).

#### Thiol-disulfide homeostasis analysis

Thiol-disulfide homeostasis parameters were measured using a commercial assay kit (Rel Assay Diagnostics, Turkey). The method is based on the reduction of disulfide bonds (–S–S) to free thiol groups (–SH) with sodium borohydride (NaBH₄). To prevent interference and excessive reduction in the DTNB (5,5′-dithiobis-[2-nitrobenzoic acid]) reaction, excess sodium borohydride (NaBH₄) was neutralized with formaldehyde. Total thiol (TT) levels were determined by the DTNB reaction, encompassing both reduced and native thiol (NT) groups. Disulfide (DIS) concentration was calculated using the formula DIS = (TT − NT)/2. DIS/TT, DIS/NT, and NT/TT ratios were also calculated to assess the thiol-disulfide balance.

#### Measurement of IL-1β, IL-6, TNF-α, TRPM4, and SUR1 levels

Interleukin-1 beta (IL-1β; MyBioSource: MBS2023030), interleukin-6 (IL-6; MyBioSource: MBS824725), tumor necrosis factor-alpha (TNF-α; MyBioSource: MBS824520), transient receptor potential cation channel M subgroup member 4 (TRPM4; MyBioSource: MBS2019135), and sulfonylurea receptor 1 (SUR1; MyBioSource: MBS1603836) levels were measured quantitatively using commercial ELISA kits. All analyses were performed in accordance with the manufacturer’s protocols. A standard curve was generated for each cytokine and appropriate volumes of standard solutions, samples and antibody-containing reagents were added to the wells. Following the specified incubation periods, wash steps were applied. Optical density values were measured at 450 nm using a BioTek Synergy™ HTX Multi-Mode Reader (BioTek Instruments, USA). Analytical reliability was ensured by keeping intra-assay and inter-assay coefficients of variation below 10%.

### Histopathological examinations

#### Histological preparation and routine staining procedures

Paraffin-embedded coronal brain tissues were sectioned at 5 µm thickness using a rotary microtome (Leica RM2235, Leica Biosystems, Germany). Sections were carefully mounted on poly-L-lysine-coated glass slides and then dried overnight at 37 °C to ensure optimal adhesion. Deparaffinization was performed by immersing the slides in xylene solution twice for 10 min each to completely remove the paraffin. Rehydration was performed with a stepwise ethanol series, starting with 100% ethanol (two changes, 5 min each), followed by 96% ethanol (5 min), 70% ethanol (5 min), and finally rinsing in distilled water for 5 min to ensure complete hydration. For general histological evaluation, hematoxylin and eosin (H&E) staining was performed to visualize general tissue structure and cellular morphology. Following staining, slides were dehydrated in a reverse ethanol series (70%, 96%, 100%), cleared in xylene, and coverslipped using Distyrene, Plasticizer, and Xylene (DPX) mounting medium (Sigma-Aldrich, USA).

#### Histopathological evaluation

Histopathological evaluation was performed under light microscopy by an investigator blinded to the experimental groups. Apoptotic neurons were identified by characteristic morphological features including cell shrinkage, eosinophilic cytoplasm, chromatin condensation, and apoptotic body formation. Degenerative changes were defined by cytoplasmic vacuolization, loss of Nissl substance, and nuclear pyknosis. Necrosis was characterized by cellular swelling, membrane disruption, karyolysis, and associated inflammatory response. Congestion was identified by dilated blood vessels filled with erythrocytes. Inflammation was assessed by the presence of perivascular or parenchymal inflammatory cell infiltration. Hemorrhage was defined as the presence of extravasated erythrocytes within the tissue parenchyma.

Neurons undergoing degeneration, necrosis, or apoptosis in the cortex and hippocampus were counted in three randomly selected non-overlapping fields per section per rat, while dentate gyrus neurons were evaluated in two fields due to anatomical constraints. Congestion, inflammation, and hemorrhage were evaluated semi-quantitatively in the same fields. Severity was graded as mild (1, focal involvement affecting < 25% of the field), moderate (2, involvement of 25–50%), and severe (3, involvement of > 50%).

#### Immunohistochemical staining for 8-hydroxy-2′-deoxyguanosine

To detect oxidative DNA damage, immunohistochemical staining for 8-hydroxy-2′-deoxyguanosine (8-OHdG) was performed on 5-µm-thick deparaffinized paraffin-embedded coronal brain sections. Antigen retrieval was achieved by incubating the sections in 10 mM citrate buffer (pH 6.0) in a water bath at 95–98 °C for 20 min. Following antigen retrieval, the sections were allowed to cool to room temperature and washed in phosphate-buffered saline (PBS, pH 7.4) for 5 min. Endogenous peroxidase activity was quenched by incubating the sections in 3% hydrogen peroxide (H₂O₂) in methanol for 10 min. Nonspecific binding sites were blocked by incubating sections with 5% normal goat serum (Vector Laboratories) in PBS containing 0.1% Triton X-100 for 1 h at room temperature. Sections were then incubated with primary antibody against 8-OHdG (sc-66036, Santa Cruz, Texas, USA) overnight at 4 °C. Following primary antibody incubation, slides were incubated with secondary antibody (Alexa 488, ab150113, Cambridge, UK). After washing, sections were mounted with a mounting medium containing Hoechst 33,342: PBS:Glycerol (1:1) (Thermo Scientific, Massachusetts, USA). Sections subjected to the same procedure without primary antibody incubation served as negative controls. All stained sections were examined and analyzed using a fluorescence microscope (Axio Vert.A1; Carl Zeiss Microscopy GmbH, Jena, Germany) equipped with a digital camera (Axiocam 503 mono; Carl Zeiss Microscopy GmbH, Jena, Germany). Images were acquired using ZEN imaging software (Zeiss) under consistent illumination and exposure settings for all samples.

Fluorescent images were analyzed using ImageJ (Fiji) software (National Institutes of Health, USA) to quantify cells showing 8-OHdG immunoreactivity. Images were separated into separate channels (Hoechst, blue; 8-OHdG, green), converted to 8-bit grayscale, and properly contrast-enhanced. A consistent thresholding method was applied to create binary masks for each channel. Hoechst staining was used to define cell boundaries and ensure that 8-OHdG signals were associated with individual cells. Colocalization of 8-OHdG with Hoechst-labeled cells was determined using the Image Calculator function, and overlapping particles were quantified. Five randomly selected fields were analyzed for each tissue section, and each field was processed three times to minimize technical variability and ensure reproducibility. Data are expressed as the mean number of 8-OHdG-positive cells per section (Snyder et al. 2017).

### Molecular studies

#### Tissue sample collection

Cervical dislocation was preferred for tissue sacrifice due to its advantages such as rapid death and the absence of chemical residue. The right hemisphere forebrain lobe was removed into Eppendorf tubes and stored at − 80 °C until the study was conducted.

#### miRNA isolation from tissues and cDNA synthesis

miRNA was isolated from the hippocampus brain tissue using the miRNeasy Micro Kit (Qiagen, Germany Catalog No: 217084). RNA concentration and purity were measured using a spectrophotometer (DENOVIX DS-11 FX, USA) on the A260 and A280. The miRCURY LNA RT Kit (Qiagen, Germany Catalog No. 339340) was used for cDNA (Complementary DNA) synthesis. Kit components were stored at − 20 °C until the study was completed, and tissue RNA samples were stored at − 80 °C. Before starting the study, kit components and RNA samples were thawed on ice. RNA concentrations were equalized to 100 ng/μL.

#### qRT-PCR analysis

Quantitative Real-Time Polymerase Chain Reaction (qRT-PCR) was performed on the Roche LightCycler® 480-II instrument using the miRCURY LNA SYBR Green PCR Kit (Qiagen, Germany Catalog No. 339346) in triplicate according to the kit protocol. Before the qRT-PCR synthesis reaction, cDNA control PCR was performed to determine the purity and concentration of complementary DNA (cDNA) samples. U6snRNA was used as the housekeeping gene. For real-time PCR, designed specific primers (hsa-miR-23a-3p, hsa-miR-34a-5p, hsa-miR-132-3p, hsa-miR-134-5p, mmu-miR-324-5p) were used. Thermal cycling conditions were 95 °C for 2 min, followed by 45 cycles of 95 °C for 10 s and 56 °C for 1 min. PCR specificity was confirmed by melting curve analysis.

Regarding miRNA analysis, data obtained were analyzed using an online software program developed by the Qiagen GeneGlobe Data Analysis Center (https://geneglobe.qiagen.com/us/analyze) specifically designed for miRCURY LNA miRNA PCR Panels and Assays. Expression levels of each miRNA were assessed using the Ct (Threshold Cycle) value. These results were normalized (ΔCt) using the housekeeping gene (U6snRNA) for each sample. Fold changes between groups were calculated as ΔΔCt. When groups were compared, differential expression of miRNA genes was expressed as 2 − ΔΔCt (Fold Regulation; FR). *p* values were calculated using the Student *t*-test for repeated 2 − ΔΔCt values for each miRNA in the control and experimental groups, and *p* values less than 0.05 were considered statistically significant. The *p*-value calculation used was based on a parametric, two-sample, equal-variance, two-tailed distribution.

### Statistical analysis

Data obtained from the experimental study were statistically analyzed using SPSS data analysis software. First, the Shapiro–Wilk test was used to determine whether the data were normally distributed. Data not showing a normal distribution were subjected to Kruskal–Wallis analysis of variance, a non-parametric analysis of variance, followed by pairwise group comparisons using the Mann–Whitney *U* test with Bonferroni correction. Data are expressed as mean ± standard error in the text and graphs. Taking two independent variables (tocopherol treatment and seizure status) into account, comparisons among the three experimental groups were limited to the control vs. TBI + PTZ groups and the TBI + PTZ vs. TBI + PTZ + tocopherol groups. Following Bonferroni correction for these two planned comparisons, statistical significance was set at *p* < 0.025 (0.05/2). H(df) and *p*-values obtained from the Kruskal–Wallis test for the significant parameters are also given.

## Results

Animals were weighed at the beginning of the experiment and on the sixth day, the final day of the experiment. No significant difference was detected between the average body weights of the animals in the TBI + PTZ group and the TBI + PTZ + tocopherol group (Fig. 1B).

### Epileptic seizure scoring

Comparing behavioral seizure scores, a significant decrease in seizures of grade 4 and 5 on the Racine scale was observed in the TBI + PTZ + tocopherol group compared to the TBI + PTZ group (*p* = 0.024), and seizure frequency and total seizure duration decreased significantly (*p* = 0.021 and *p* = 0.016, respectively; Fig. 2A–D). There was no significant difference in the total amount of PTZ administered to induce seizures in the TBI + PTZ + tocopherol group compared to the TBI + PTZ group (Fig. 2E). Since statistical analyses regarding epileptic seizure scoring were performed between the TBI + PTZ and TBI + PTZ + tocopherol groups, only *p*-values obtained from the Mann Whitney *U* test are presented.

### Behavioral test data

**Fig. 3:**
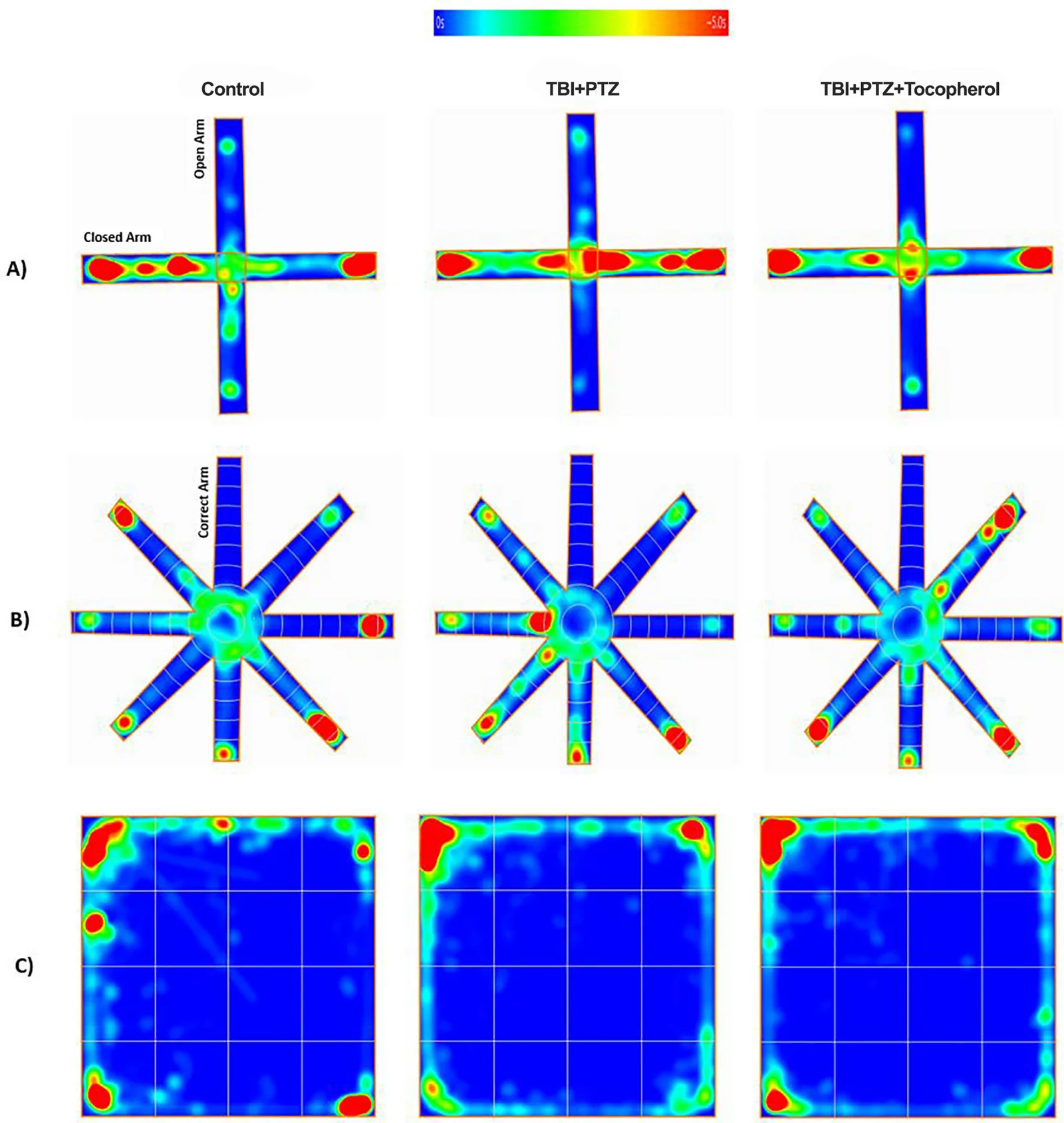
Behavioral test heat map. Represents the areas where the animals roamed in the test setup. **A** Elevated plus maze. **B** Radial arm maze. **C** Open field test. Note: Control group (*n* = 8); TBI + PTZ group (*n* = 10); TBI + PTZ + Tocopherol group (*n* = 10)

**Fig. 4:**
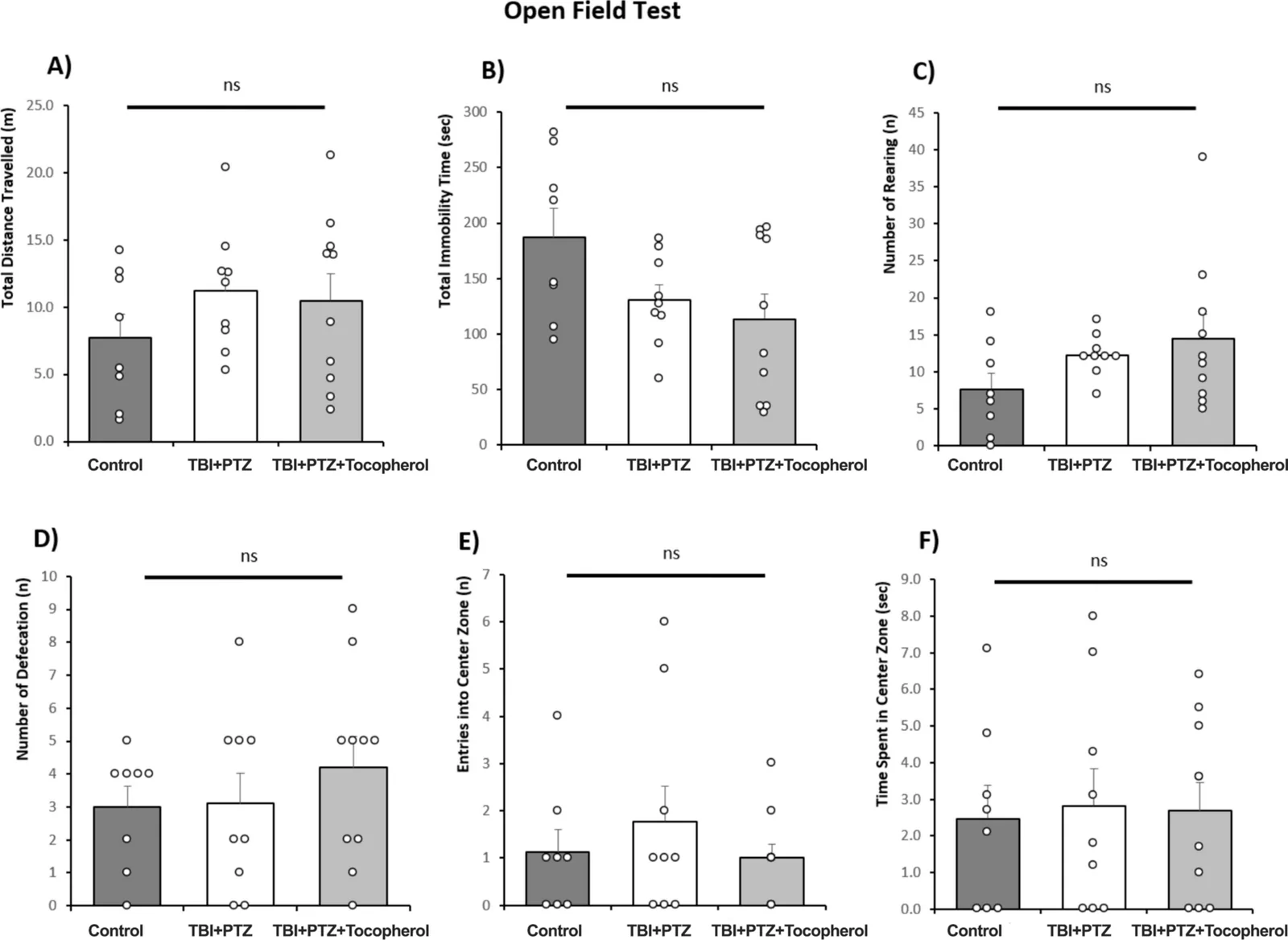
Open field test parameters. **A** Total distance traveled. **B** Total immobility time. **C** Number of rearings. **D** Number of defecations. **E** Number of entries into the central squares. **F** Total time spent in the central squares. Note: Control group (*n* = 8); TBI + PTZ group (*n* = 10); TBI + PTZ + Tocopherol group (*n* = 10). Data are given with mean ± SEM. Statistical analyses were performed after the Kruskal-Walli’s analysis of variance, using the Bonferroni-corrected Mann–Whitney *U* post hoc test for pairwise group comparisons: a *p*-value < 0.025 (0.05/2 = 0.025) was considered statistically significant

A heat map was created showing the locations of animals in the open field, radial arm maze, and elevated plus maze test (Fig. 3). In the open field test, there were no significant differences in the TBI + PTZ group compared to the control group, nor in the TBI + PTZ + tocopherol group compared to the TBI + PTZ group in terms of total distance travelled, total immobility time, number of rearing, number of defecations, number of central area entries, and time spent in the central area (Fig. 4).

**Fig. 5:**
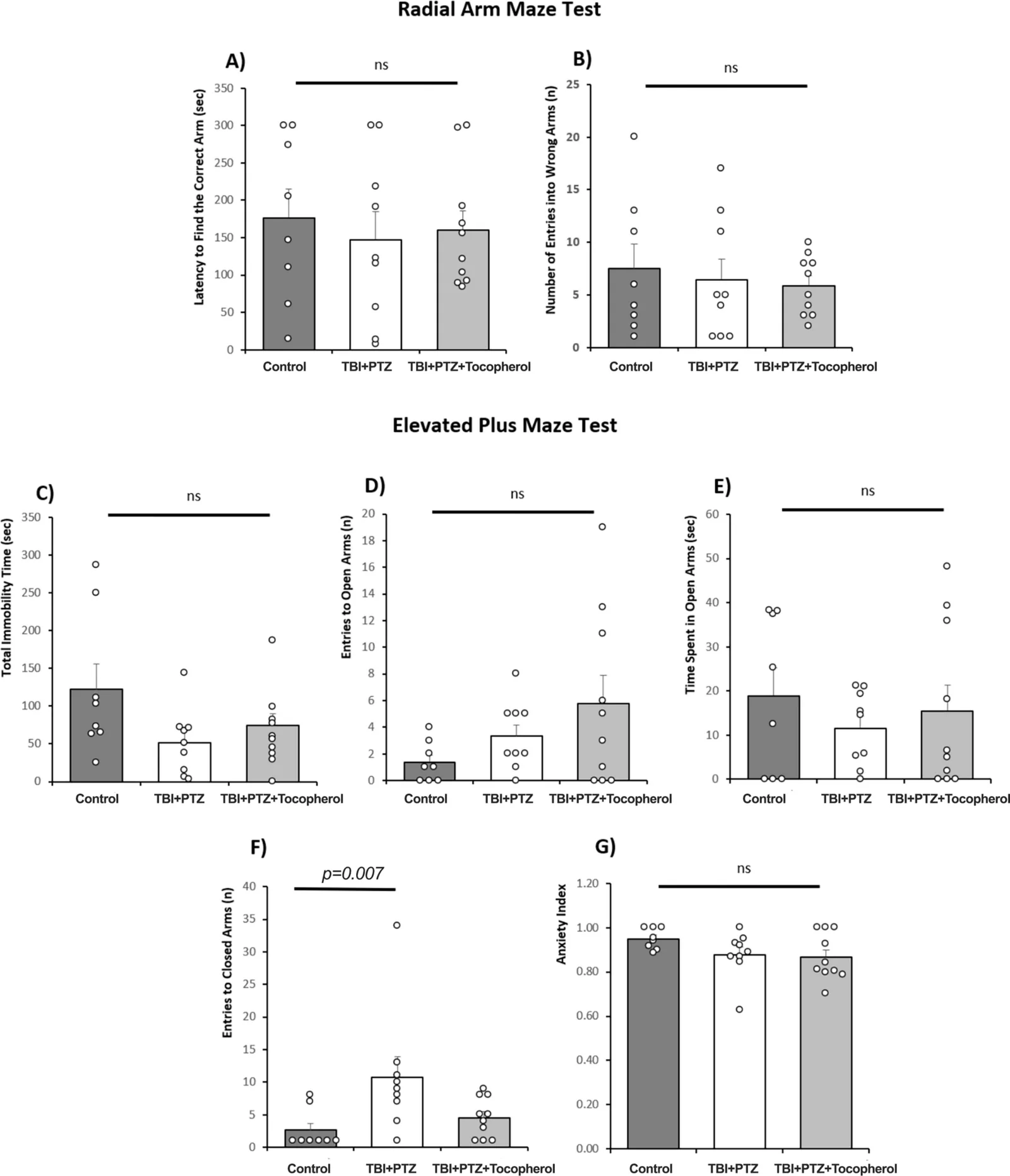
Radial arm maze (**A**–**C**) and elevated plus maze (**D**–**G**) test results. **A** Latency to find the correct arm. **B** Number of errors (entries into incorrect arms). **C** Total immobility duration. **D** Number of entries into open arms. **E** Time spent in open arms. **F** Number of entries into closed arms. **G** Anxiety index. Note: Control group (*n* = 8); TBI + PTZ group (*n* = 10); TBI + PTZ + Tocopherol group (*n* = 10). Data are given with mean ± SEM. Statistical analyses were performed after Kruskal-Walli’s analysis of variance, using the Bonferroni-corrected Mann–Whitney *U* post hoc test for pairwise group comparisons: a *p*-value < 0.025 (0.05/2 = 0.025) was considered statistically significant

In the radial arm maze test, when the latency to find the correct arm, the number of incorrect arm entries, and the total immobility time were compared, there were no significant differences in the TBI + PTZ group compared to the control group, nor in the TBI + PTZ + tocopherol group compared to the TBI + PTZ group (Fig. 5A–C). In the elevated plus maze test, no significant difference was observed between the groups in terms of total immobility time, entries to open arms, time spent in the open arm and anxiety index (Fig. 5C–E and G). However, the number of closed arm entries showed a significant difference in the Kruskal–Wallis analysis (H(2) = 9.174, *p* = 0.01). Compared to the control group, the number of closed arm entries was found to be significantly reduced in the TBI + PTZ group (*p* = 0.007; Fig. 5F).

### Serum biochemical analysis data

SUR 1-TPRM 4 Cation channel values: SUR1 showed a significant difference in the Kruskal–Wallis (KW) analysis (H(2) = 23.362, *p* = 0.000). SUR 1 levels were found to be significantly increased in the TBI + PTZ group compared to the control group (*p* = 0.000) and significantly decreased in the TBI + PTZ + tocopherol group compared to the TBI + PTZ group (*p* = 0.000; Table 1). When TPRM4 levels were compared (KW: H(2) = 19.578, *p* = 0.000), it was found to be increased in the TBI + PTZ group compared to the control group (*p* = 0.000) and decreased in the TBI + PTZ + tocopherol group compared to the TBI + PTZ group (*p* = 0.016; Table 1).

**Table 1:** Comparison of serum oxidative stress markers, inflammatory cytokines, and thiol/disulfide homeostasis among Control, TBI + PTZ, and TBI + PTZ + Tocopherol groups

	Control	TBI + PTZ	TBI + PTZ + Tocopherol	*KW: H(df), p-value*	*MW: p-values*
Sulfonylurea receptor 1 (ng/ml)	0.58 ± 0.05	3.88 ± 0.19 *	1.94 ± 0.18 ^***&***^	*H(2)* = *23.362, p* = *0.000*	*0.000; ^***&***^*0.000*
Transient receptor potential cation channel subfamily M member 4 (ng/ml)	0.26 ± 0.03	2.54 ± 0.23 *	1.77 ± 0.16 ^***&***^	*H(2)* = *19.578, p* = *0.000*	*0.000; ^***&***^*0.016*
Interleukin-1 beta (pg/ml)	117 ± 7	557 ± 30 *	394 ± 27 ^***&***^	*H(2)* = *22.017, p* = *0.000*	*0.000; ^***&***^*0.001*
Interleukin-6 (pg/ml)	258 ± 10	1111 ± 47 *	645 ± 22 ^***&***^	*H(2)* = *23.941, p* = *0.000*	*0.000; ^***&***^*0.000*
Tumor necrosis factor-alpha (pg/ml)	52 ± 4	457 ± 23 *	252 ± 1^***&***^	*H(2)* = *23.941, p* = *0.000*	*0.000; ^***&***^*0.000*
Total oxidant status (Eq/L)	6 ± 0.31	14 ± 0.71 *	10 ± 0.20 ^***&***^	*H(2)* = *23.941, p* = *0.000*	*0.000; ^***&***^*0.000*
Total antioxidant status (Eq/L)	0.51 ± 0.02	0.15 ± 0.01 *	0.29 ± 0.02 ^***&***^	*H(2)* = *23.941, p* = *0.000*	*0.000; ^***&***^*0.000*
Oxidative stress index	13 ± 0.83	100 ± 6 *	37 ± 2 ^***&***^	*H(2)* = *23.941, p* = *0.000*	*0.000; ^***&***^*0.000*
Total thiol (µmol/L)	418 ± 12	244 ± 6 *	331 ± 14 ^***&***^	*H(2)* = *21.748, p* = *0.000*	*0.000; ^***&***^*0.001*
Native thiol (µmol/L)	389 ± 9	116 ± 4 *	271 ± 12 ^***&***^	*H(2)* = *23.941, p* = *0.000*	*0.000; ^***&***^*0.000*
Disulfide concentrations (µmol/L)	16 ± 1.32	63 ± 2.37 *	30 ± 5.55 ^***&***^	*H(2)* = *18.927, p* = *0.000*	*0.000; ^***&***^*0.002*
Native thiol/total thiol (%)	93 ± 1.08	47 ± 1.53 *	82 ± 2.75 ^***&***^	*H(2)* = *23.422, p* = *0.000*	*0.000; ^***&***^*0.000*
Disulfide/total thiol (%)	3 ± 0.54	26 ± 0.77 *	8 ± 1.37 ^***&***^	*H(2)* = *23.422, p* = *0.000*	*0.000; ^***&***^*0.000*
Disulfide/native thiol (%)	4 ± 0.32	55 ± 3.38 *	11 ± 2.53 ^***&***^	*H(2)* = *23.422, p* = *0.000*	*0.000; ^***&***^*0.000*

Control (*n* = 8); TBI + PTZ (*n* = 10); TBI + PTZ + Tocopherol (*n* = 10). Data are given with mean ± SEM. Statistical analyses were performed after the Kruskal-Walli’s (KW) analysis of variance, using the Bonferroni-corrected Mann–Whitney *U* (MW) post hoc test for pairwise group comparisons: a *p*-value < 0.025 (0.05/2 = 0.025) was considered statistically significant

*TBI+PTZ group compared to control group. & TBI+PTZ+Tocopherol group compared to TBI+PTZ group

Inflammatory Cytokine Response: When interleukin-1 beta (KW: H(2) = 22.017, *p* = 0.000), interleukin-6 (KW: H(2) = 23.941, *p* = 0.000), and tumor necrosis factor-alpha (KW: H(2) = 23.941, *p* = 0.000) levels were compared, it was observed that the TBI + PTZ group values were significantly increased compared to the control group (*p* = 0.000), while the TBI + PTZ + tocopherol group values were significantly decreased compared to the TBI + PTZ group (p = 0.001, *p* = 0.000, *p* = 0.000, respectively; Table 1).

Oxidative Stress and Antioxidant Capacity: When oxidative stress values were compared (all three of them, KW: H(2) = 23.941, *p* = 0.000), it was observed that the TOS and OSI values in the TBI + PTZ group increased compared to the control group (*p* = 0.000), while the TAS value decreased (*p* = 0.000). The TOS and OSI values in the TBI + PTZ + tocopherol group increased compared to the TBI + PTZ group (*p* = 0.000), while the TAS value decreased (*p* = 0.000; Table 1).

Thiol-Disulfide Homeostasis: Total thiol, native thiol, and disulfide concentrations showed significant differences in the KW variance analysis (H(2) = 21.748, *p* = 0.000; H(2) = 23.941, *p* = 0.000; H(2) = 18.927, *p* = 0.000, respectively). Compared to the control group, total thiol and native thiol levels were found to be decreased (*p* = 0.000), while disulfide concentration was increased (*p* = 0.000) in the TBI + PTZ group. When the TBI + PTZ + tocopherol group was compared to the TBI + PTZ group, total thiol and native thiol levels were found to be increased (*p* = 0.001 and *p* = 0.000, respectively), while disulfide concentration was found to be decreased (*p* = 0.002; Table 1). %Native Thiol/Total Thiol, % Disulfide/Total Thiol ratio and % Disulfide/Native Thiol ratios showed significant differences in the KW variance analysis (all three of them, H(2) = 23.422, *p* = 0.000, *p* = 0.000). The %Native Thiol/Total Thiol ratio was found to be decreased in the TBI + PTZ group compared to the control group (*p* = 0.000) and increased in the TBI + PTZ + tocopherol group compared to the TBI + PTZ group (*p* = 0.000; Table 1). When the % Disulfide/Total Thiol ratio and % Disulfide/Native Thiol ratio were compared, it was found that the TBI + PTZ group values increased significantly compared to the control group (*p* = 0.000), and the TBI + PTZ + tocopherol group values decreased significantly compared to the TBI + PTZ group (*p* = 0.000; Table 1).

### Biochemical analysis data in brain tissue

**Fig. 6:**
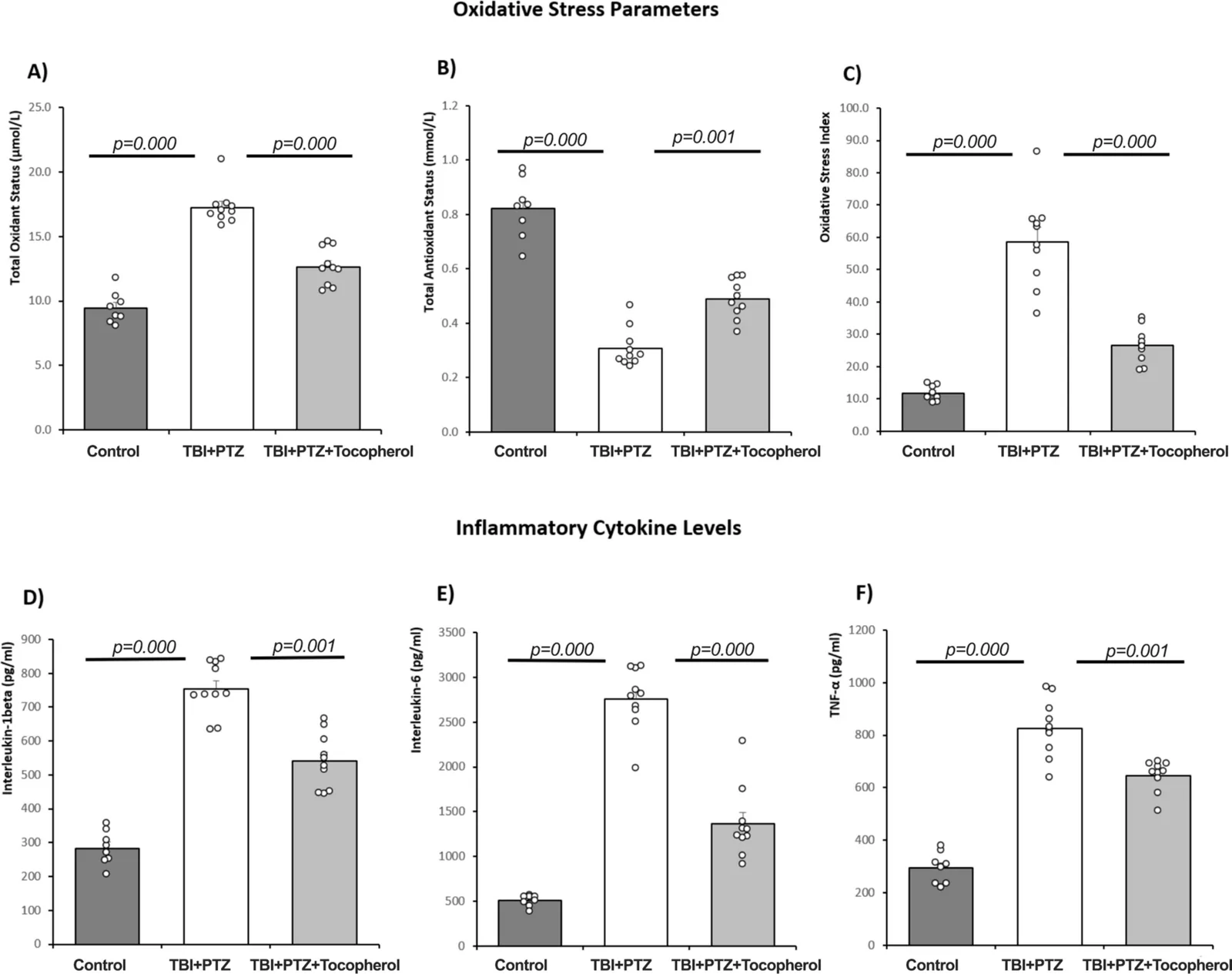
Oxidative stress and inflammatory cytokine levels. **A** TOS levels increased significantly in the TBI + PTZ group compared with the Control group and TBI + PTZ + Tocopherol group. **B** TAS levels were significantly lower in the TBI + PTZ group compared with the Control group and TBI + PTZ + Tocopherol group. **C** OSI increased significantly in the TBI + PTZ group but decreased after tocopherol treatment. **D** IL-1β levels were elevated in the TBI + PTZ group but significantly reduced in the TBI + PTZ + Tocopherol group. **E** IL-6 levels were significantly higher in the TBI + PTZ group and lower in the TBI + PTZ + Tocopherol group. **F** TNF-α levels increased in the TBI + PTZ group and decreased significantly following tocopherol treatment. Note: TAS: total antioxidant status; TOS: total oxidant status; OSI: oxidative stress index; IL-1β: interleukin-1β; IL-6: interleukin-6; TNF-α: tumor necrosis factor-α. Control group (*n* = 8); TBI + PTZ group (*n* = 10); TBI + PTZ + Tocopherol group (*n* = 10). Data are given with mean ± SEM. Statistical analyses were performed after the Kruskal-Walli’s analysis of variance, using the Bonferroni-corrected Mann–Whitney *U* post hoc test for pairwise group comparisons: a *p*-value < 0.025 (0.05/2 = 0.025)) was considered statistically significant

Oxidative Stress and Antioxidant Capacity: Brain TOS, TAS, and OSI showed a significant difference in the KW analysis (H(2) = 23.173, *p* = 0.000; H(2) = 22.537, *p* = 0.000; H(2) = 23.941, *p* = 0.000, respectively). TOS levels were found to be significantly increased in the TBI + PTZ group compared to the control group (*p* = 0.000), while they were significantly decreased in the TBI + PTZ + tocopherol group compared to the TBI + PTZ group (*p* = 0.000; Fig. 6A). TAS values in the TBI + PTZ group were found to be significantly decreased compared to the control group (*p* = 0.000), while TAS values in the TBI + PTZ + tocopherol group increased compared to the TBI + PTZ group (*p* = 0.001; Fig. 6B). Oxidative stress index values were found to be significantly increased in the TBI + PTZ group compared to the control group (*p* = 0.000), while they were significantly decreased in the TBI + PTZ + tocopherol group compared to the TBI + PTZ group (*p* = 0.000; Fig. 6C).

Inflammatory Cytokine Response: Brain IL-1β, IL-6, and TNF-α levels showed a significant difference in the KW analysis (H(2) = 22.806, *p* = 0.000; H(2) = 23.648, *p* = 0.000; H(2) = 22.017, *p* = 0.000, respectively). IL-1β levels were significantly increased in the TBI + PTZ group compared to the control group (*p* = 0.000), while they were significantly decreased in the TBI + PTZ + tocopherol group compared to the TBI + PTZ group (*p* = 0.001; Fig. 6D). When IL-6 levels were compared, they were significantly increased in the TBI + PTZ group compared to the control group (*p* = 0.000) and significantly decreased in the TBI + PTZ + tocopherol group compared to the TBI + PTZ group (*p* = 0.000; Fig. 6E). When the groups were compared in terms of TNF-α levels, they were found to be increased in the TBI + PTZ group compared to the control group (*p* = 0.000) and decreased in the TBI + PTZ + tocopherol group compared to the TBI + PTZ group (*p* = 0.001; Fig. 6F).

**Fig. 7:**
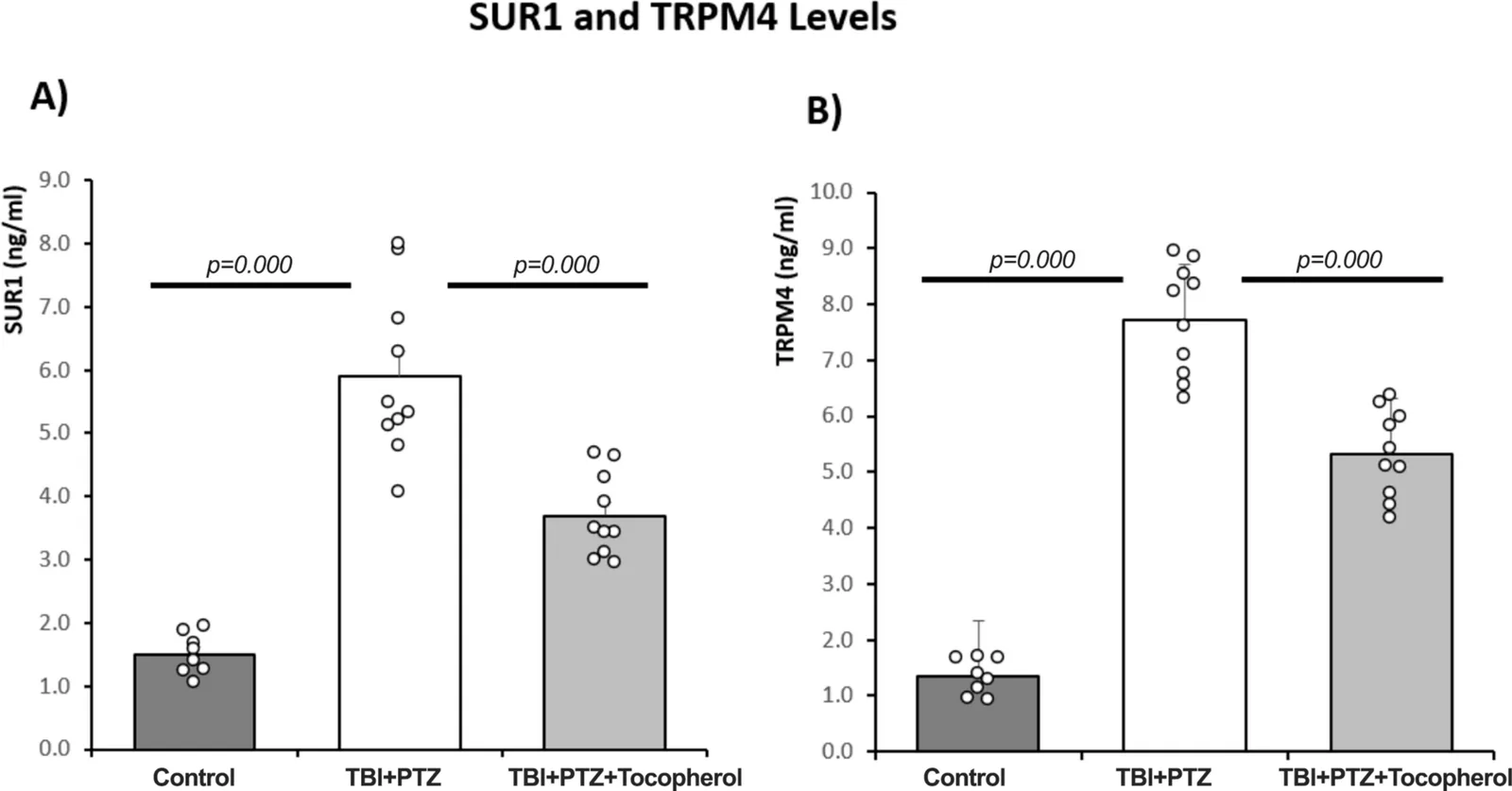
SUR1 and TRPM4 levels. **A** SUR1 levels were significantly higher in the TBI + PTZ group compared to both the control and TBI + PTZ + Tocopherol groups. **B** TRPM4 levels were significantly increased in the TBI + PTZ group and reduced after tocopherol treatment. Note: SUR1: sulfonylurea receptor 1; TRPM4: transient receptor potential melastatin 4. Control group (*n* = 8); TBI + PTZ group (*n* = 10); TBI + PTZ + Tocopherol group (*n* = 10). Data are given with mean ± SEM. Statistical analyses were performed after Kruskal-Walli’s analysis of variance, using the Bonferroni-corrected Mann–Whitney *U* post hoc test for pairwise group comparisons: a *p*-value < 0.025 (0.05/2 = 0.025) was considered statistically significant

SUR1 and TPRM4 values: Brain SUR1 and TPRM4 levels showed a significant difference in the KW analysis (H(2) = 23.081, *p* = 0.000; H(2) = 23.648, *p* = 0.000; respectively). When SUR1 values were compared, a significant increase was observed in the TBI + PTZ group compared to the control group (*p* = 0.000), while a significant decrease was observed in the TBI + PTZ + tocopherol group compared to the TBI + PTZ group (*p* = 0.000; Fig. 7A). TRPM4 levels were significantly higher in the TBI + PTZ group compared to the control group (*p* = 0.000), but significantly lower in the TBI + PTZ + tocopherol group compared to the TBI + PTZ group (*p* = 0.000; Fig. 7B).

### Histopathological examinations

**Fig. 8:**
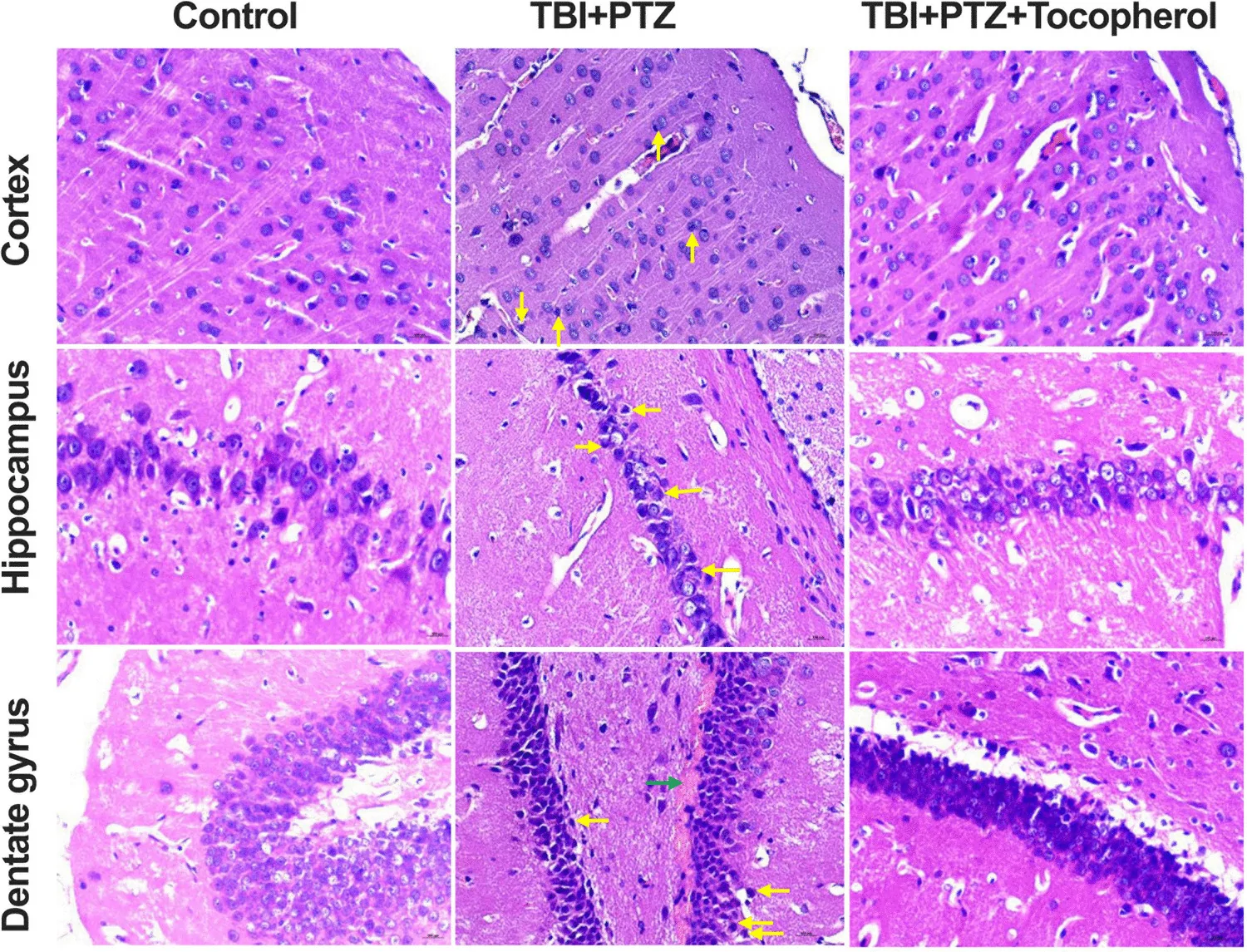
Representative histological images showing apoptosis/single-cell necrosis, neuronal degeneration (yellow arrows), and congestion (green arrows) in the superficial cerebral cortex, hippocampus, and dentate gyrus of rats following traumatic brain injury. Left panel: Control rat, showing few or no affected cells across the cortex, hippocampus, and dentate gyrus. Middle panel: Traumatic brain injury model rat, with affected neurons displaying degenerative and apoptotic features, including hypereosinophilic cytoplasm, chromatin condensation, and pericellular clearing; classical apoptotic bodies with fragmented nuclei and cytoplasmic blebs were rare. Right panel: Tocopherol-treated rat, showing a limited number of affected cells throughout the cortex, hippocampus, and dentate gyrus. Staining: H&E; original magnification: 10 ×

Representative histological images showing apoptosis/single-cell necrosis and neuronal degeneration (yellow arrows) in the superficial cerebral cortex, hippocampus, and dentate gyrus of rats following traumatic brain injury are presented in Fig. 8. The TBI + PTZ group had affected neurons exhibiting degenerative and apoptotic features such as hypereosinophilic cytoplasm, chromatin condensation, and pericellular clearing. Classic apoptotic bodies with fragmented nuclei and cytoplasmic blebs were rare. In the TBI + PTZ + tocopherol group, a limited number of affected cells were seen throughout the cortex, hippocampus, and dentate gyrus (Fig. 8).

The cortex was examined and scored for degeneration, necrosis, apoptosis, congestion, inflammation, and hemorrhage. Cortical degeneration, necrosis, and congestion showed a significant difference in the KW analysis (H(2) = 29.076, *p* = 0.000; H(2) = 28.973, *p* = 0.000; H(2) = 10.427, *p* = 0.005, respectively). Compared to the control group, degeneration, necrosis, and congestion values were significantly increased in the TBI + PTZ group (*p* = 0.003, *p* = 0.000, and *p* = 0.002, respectively), while no significant difference was found in apoptosis, inflammation, and hemorrhage values. When the TBI + PTZ group is compared with the TBI + PTZ + tocopherol group, degeneration and necrosis values were significantly decreased (*p* = 0.000 and *p* = 0.000, respectively), and no significant difference was found in apoptosis, congestion, inflammation, and hemorrhage values (Table 2).

**Table 2:** Histopathological scores in the superficial cerebral cortex, hippocampus, and dentate gyrus of rats after traumatic brain injury

	Control	TBI + PTZ	TBI + PTZ + Tocopherol	*KW: H(df), p-value*	*MW: p-values*
Cortex degeneration	0.8333 ± 0.17	2.000 ± 0.27 *	0.2333 ± 0.09 ^***&***^	*H(2)* = *29.076, p* = *0.000*	*0.003; ^***&***^*0.000*
Cortex necrosis	0.0833 ± 0.06	0.900 ± 0.15 *	0.0667 ± 0.05 ^***&***^	*H(2)* = *28.973, p* = *0.000*	*0.000; ^***&***^*0.000*
Cortex apoptosis	0.1667 ± 0.08	0.2333 ± 0.08	0.1000 ± 0.06	*ns*	*ns*
Cortex congestion	0.2500 ± 0.09	0.9333 ± 0.16 *	0.5667 ± 0.13	*H(2)* = *10.427, p* = *0.005*	*0.002
Cortex inflammation	0.125 ± 0.07	0.2000 ± 0.12	0.1000 ± 0.06	*ns*	*ns*
Cortex hemorrhage	0.0000 ± 0.00	0.0333 ± 0.03	0.0000 ± 0.00	*ns*	*ns*
Hippocampus degeneration	0.6667 ± 0.16	1.4333 ± 0.23	0.2333 ± 0.09 ^***&***^	*H(2)* = *19.481, p* = *0.000*	^***&***^* 0.000*
Hippocampus necrosis	0.1250 ± 0.07	0.6667 ± 0.15 *	0.1000 ± 0.06 ^***&***^	*H(2)* = *14.800, p* = *0.001*	*0.005; ^***&***^* 0.001*
Hippocampus apoptosis	0.1250 ± 0.07	0.1333 ± 0.08	0.1000 ± 0.06	*ns*	*ns*
Hippocampus congestion	0.2083 ± 0.08	0.5667 ± 0.11	0.3333 ± 0.10	*ns*	*ns*
Hippocampus inflammation	0.1250 ± 0.07	0.2000 ± 0.07	0.1000 ± 0.06	*ns*	*ns*
Hippocampus hemorrhage	0.0000 ± 0.00	0.1000 ± 0.07	0.0000 ± 0.00	*ns*	*ns*
Dentate gyrus degeneration	0.8125 ± 0.19	1.6000 ± 0.29	0.1500 ± 0.08 ^***&***^	*H(2)* = *17.036, p* = *0.000*	^***&***^*0.000*
Dentate gyrus necrosis	0.1250 ± 0.09	0.6000 ± 0.18	0.0500 ± 0.05^***&***^	*H(2)* = *9.057, p* = *0.011*	^***&***^*0.008*
Dentate gyrus apoptosis	0.1875 ± 0.14	0.2500 ± 0.12	0.1000 ± 0.07	*ns*	*ns*
Dentate gyrus congestion	0.1875 ± 0.10	1.1000 ± 0.26*	0.3500 ± 0.13	*H(2)* = *8.332, p* = *0.016*	*0.011
Dentate gyrus inflammation	0.0625 ± 0.06	0.1000 ± 0.07	0.0500 ± 0.05	*ns*	*ns*
Dentate gyrus hemorrhage	0.0000 ± 0.00	0.0000 ± 0.00	0.0000 ± 0.00	*ns*	*ns*

Control (*n* = 8); TBI + PTZ (*n* = 10); TBI + PTZ + Tocopherol (*n* = 10). Data are given with mean ± SEM. Statistical analyses were performed after Kruskal-Walli’s (KW) analysis of variance. using the Bonferroni-corrected Mann–Whitney *U* (MW) post hoc test for pairwise group comparisons: a *p*-value < 0.025 (0.05/2 = 0.025) was considered statistically significant. ^*^TBI + PTZ group compared to control group. ^&^TBI + PTZ + Tocopherol group compared to PTE group. *ns*, not significant

The hippocampus, as in the cortex, was examined and scored for degeneration, necrosis, apoptosis, congestion, inflammation, and hemorrhage. Hippocampal necrosis showed a significant difference in the KW analysis (H(2) = 14.800, *p* = 0.001). Compared to the control group, degeneration and necrosis scores in the TBI + PTZ group were significantly increased (*p* = 0.005), while there was no significant difference in apoptosis, inflammation, and hemorrhage values. When the TBI + PTZ + tocopherol group was compared with the TBI + PTZ group, degeneration and necrosis values were significantly decreased (*p* = 0.000 and *p* = 0.001, respectively), while there was no significant difference in apoptosis, congestion, inflammation, and hemorrhage values (Table 2).

The dentate gyrus, like the cortex and hippocampus, was examined and scored for degeneration, necrosis, apoptosis, congestion, inflammation, and hemorrhage. Compared to the control group, the congestion score in the TBI + PTZ group was significantly increased (KW: H(2) = 8.332, *p* = 0.016; MW: *p* = 0.011), while there was no significant difference in degeneration, necrosis, apoptosis, inflammation, and hemorrhage values. When the TBI + PTZ + tocopherol group was compared with the TBI + PTZ group, degeneration and necrosis values were significantly decreased (*p* = 0.000 and *p* = 0.008, respectively), while there was no significant difference in apoptosis, congestion, inflammation, and hemorrhage values (Table 2).

### Assessment of oxidative DNA damage with 8-OHdG immunostaining

**Fig. 9:**
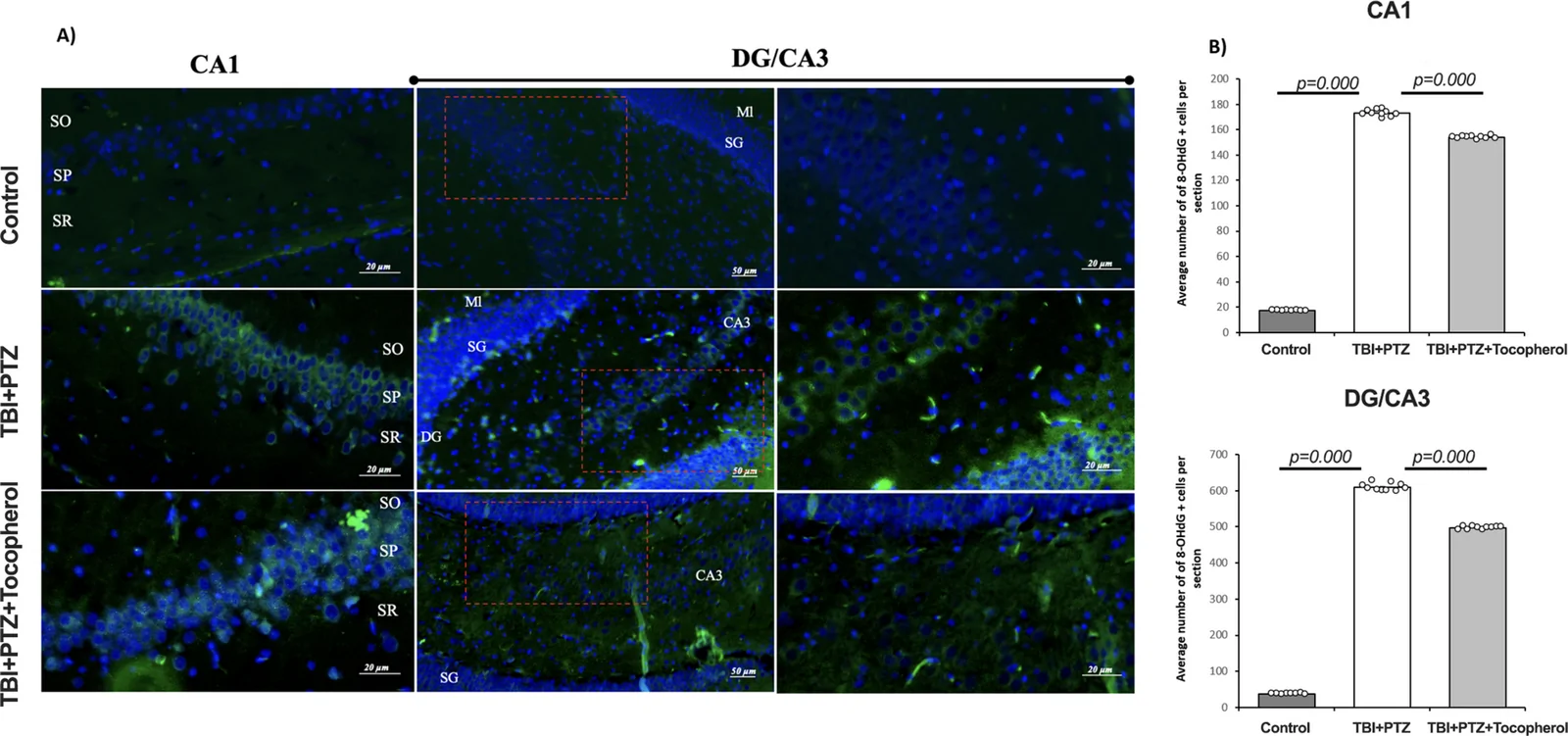
8-OHdG immunoreactivity in hippocampal regions. Representative hippocampal sections from Control, TBI + PTZ, and TBI + PTZ + Tocopherol groups stained for 8-hydroxy-2′-deoxyguanosine (8-OHdG; green, Alexa Fluor 488) to assess oxidative DNA damage and counterstained with Hoechst 33,342 (blue). **A** In CA1, 8-OHdG immunoreactivity was minimal in the Control group but markedly increased in the TBI + PTZ group, particularly in the stratum pyramidale (SP), with additional signal in the stratum oriens (SO) and stratum radiatum (SR). Tocopherol treatment reduced 8-OHdG staining. **B** Quantitative analysis of 8-OHdG-positive cells in CA1 and DG. Images: 20 × (scale = 50 µm) and 40 × (scale = 20 µm). Note: SP, stratum pyramidale; SO, stratum oriens; SR, stratum radiatum; ML, molecular layer; SG, stratum granulosum; CA1/CA3, Cornu Ammonis 1/3; DG, dentate gyrus. Control group (*n* = 8); TBI + PTZ group (*n* = 10); TBI + PTZ + Tocopherol group (*n* = 10). Data are given with mean ± SEM. Statistical analyses were performed after the Kruskal-Walli’s analysis of variance, using the Bonferroni-corrected Mann–Whitney *U* post hoc test for pairwise group comparisons: a *p*-value < 0.025 (0.05/2 = 0.025) was considered statistically significant

To assess the levels of oxidative stress associated with post-traumatic epilepsy, we performed immunofluorescence staining for 8-OHdG, a well-known marker of oxidative DNA damage, in hippocampal slices. In the control group, 8-OHdG immunoreactivity was minimal or absent in all hippocampal subregions examined, including CA1, CA3, and the dentate gyrus (DG), indicating low baseline oxidative DNA damage under normal conditions (Fig. 9). In contrast, the TBI + PTZ group exhibited strong 8-OHdG expression in all hippocampal subfields. In the CA1 region, strong cytoplasmic 8-OHdG immunoreactivity was observed primarily in the stratum pyramidale (SP), which contains pyramidal neurons. Additional staining was observed in the stratum oriens (SO) and stratum radiatum (SR), suggesting that both neuronal and glial populations are exposed to high oxidative stress in the TBI + PTZ group. Similarly, in the CA3 region, intense 8-OHdG staining was localized in the pyramidal cell layer, while in the DG, prominent signal was detected in the stratum granulosum (SG), molecular layer (MI), and hilus, further supporting widespread oxidative damage (Fig. 9A). In the alpha-tocopherol-treated TBI + PTZ + tocopherol group, 8-OHdG immunoreactivity was significantly reduced in all hippocampal subregions compared to the untreated TBI + PTZ group. In CA1, 8-OHdG staining in the SP, SO, and SR was significantly attenuated, demonstrating that tocopherol attenuates TBI and seizure-induced oxidative DNA damage. Similar decreases in 8-OHdG levels were observed in CA3 and DG, suggesting a partial protective effect of tocopherol throughout the hippocampus (Fig. 9A). Quantitative analysis supported these observations. The number of 8-OHdG-positive of CA1 and DG/CA3 showed a significant difference in the KW analysis (H(2) = 24.159, *p* = 0.000; H(2) = 24.033, *p* = 0.000, respectively). In the TBI + PTZ group, the number of 8-OHdG-positive cells per slice was increased in both CA1 and DG/CA3 regions compared to the control group (*p* = 0.000). Tocopherol treatment reduced the number of 8-OHdG-positive cells in both regions compared to the TBI + PTZ group (*p* = 0.000), confirming the protective effect (Fig. 9B). Collectively, these results suggest that post-traumatic epilepsy is associated with significant oxidative DNA damage in hippocampal neurons and glia, and that tocopherol treatment reduces this oxidative stress, highlighting its potential neuroprotective role in the context of TBI + PTZ.

### miRNA analysis

**Fig. 10:**
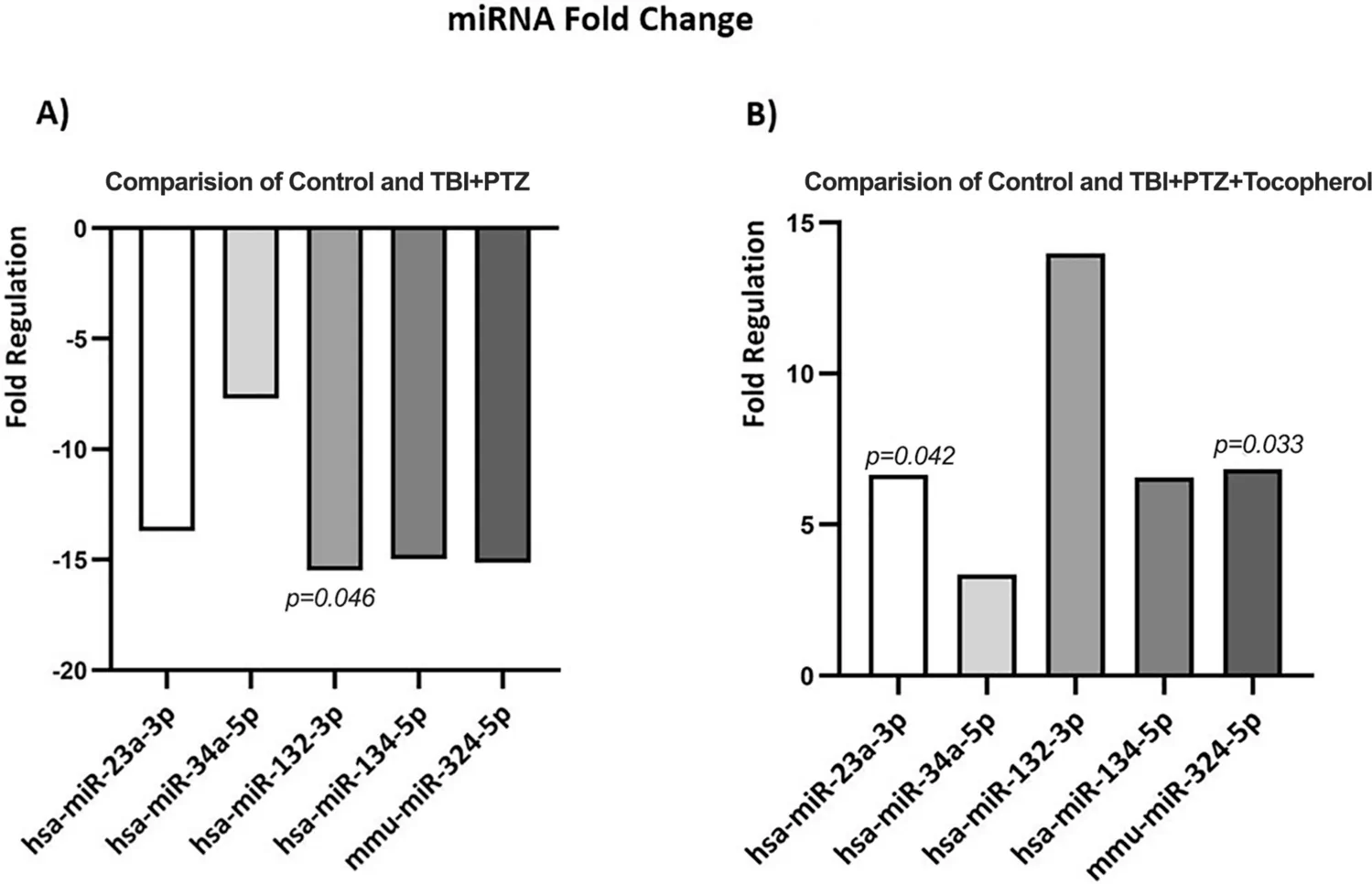
miRNA analysis. **A** Comparison between the Control and TBI + PTZ groups. **B** Comparison between the TBI + PTZ group and TBI + PTZ + Tocopherol groups. Note: Control (*n* = 8); TBI + PTZ group (*n* = 10); TBI + PTZ + Tocopherol group (*n* = 10). Fold changes between groups were calculated as ΔΔCt. When groups were compared, differential expression of miRNA genes was expressed as 2 − ΔΔCt (Fold Regulation; FR). *P* values were calculated using the Student *t*-test for repeated 2 − ΔΔCt values for each miRNA in the control and experimental groups, and *p* values less than 0.05 were considered statistically significant. The *p*-value calculation used was based on a parametric, two-sample, equal-variance, two-tailed distribution

When comparing the control group and the TBI + PTZ group, all genes (hsa-miR-23a-3p, hsa-miR-34a-5p, hsa-miR-132-3p, and hsa-miR-324-5p) were downregulated. Of these five miRNAs, the downregulation of miR-132-3p was found to be statistically significant (*p* = 0.046; Fig. 10A). When the TBI + PTZ group was compared with the TBI + PTZ + tocopherol group, all examined miRNAs were upregulated (Fig. 10B). Among these miRNAs, mir-23a-3p and miR-324-5p were found to be statistically significant based on the *p* value (*p* = 0.042 and *p* = 0.033, respectively). *p* values were calculated using the Student t-test for repeated 2 − ΔΔCt values for each miRNA in the control and experimental groups, and *p* values less than 0.05 were considered statistically significant.

## Discussion

In various experimental models, pretreatment with alpha-tocopherol and dietary antioxidants has been shown to reduce the severity and deleterious effects of seizures by reducing the formation of seizure-induced oxygen and nitrogen free radicals (Zaja-Milatovic et al. 2008; Tomé et al. 2010). Combination pretreatment with ascorbic acid, alpha-tocopherol, and sodium pyruvate, designed to synergistically target mitochondrial disorders, reduced the severity of kainic acid-induced seizures in mice, providing complete protection against severe tonic–clonic seizures (Simeone et al. 2014). Additionally, alpha-tocopherol has been reported to improve seizure control and reduce oxidative stress when used in conjunction with antiepileptic drugs in antiepileptic drug-resistant patients (Mehvari et al. 2016). In the present study, in behavioral seizure scores obtained with PTZ, alpha-tocopherol significantly reduced seizure severity, increased seizure latency (although not statistically significant), and significantly reduced seizure frequency and total seizure duration. These results are consistent with the literature regarding the effect of alpha-tocopherol on seizures. The antioxidant properties of alpha-tocopherol may mediate anti-inflammatory and neuroprotective effects in the early stages of clinical management of epilepsy. However, some effects on neuroglial and neuronal markers affected by status epilepticus were also observed in seizure-free rats. This suggests that alpha-tocopherol may affect the structural and functional properties of neurons, as well as microglial and astrocytic cells, even in the absence of abnormal oxidative stress (Ambrogini et al. 2016). Our findings demonstrate that alpha-tocopherol restored TBI-induced decreases in total antioxidant capacity and attenuated increases in total oxidant capacity and oxidative stress index in both serum and tissue samples. In the present study, total thiol and native thiol levels were significantly lower in the TBI + PTZ group compared to the control group, while disulfide levels were increased. The significant correction of this imbalance by alpha-tocopherol supports its important role in balancing thiol-disulfide homeostasis, a critical balance for cellular repair processes (Velat et al. 2011). This finding is consistent with the literature showing that agents that restore the disrupted thiol-disulfide balance can improve outcomes in diseases caused by oxidative stress (Li et al. 2019).

In recent years, studies conducted with animal models have shown that antioxidant compounds, including vitamin E, can ameliorate or prevent the decrease in hippocampal long-term potentiation (LTP) in mammalian learning and memory (Malenka and Nicoll 1999)and the impairment in hippocampus-dependent cognitive abilities that occurs under various conditions associated with increased oxidative stress (Ambrogini et al. 2016). Adult neurogenesis is an important form of lifelong neural plasticity. It consists of a multistep process, resulting in the formation of new neurons, which in turn contribute to neural plasticity. It has great potential for repairing the diseased or aging brain (Herrera-Arozamena et al. 2016). It has also been reported to play a significant role in the development of anxiety disorders, depression, and age-related deficits (Jinno 2016). It is also considered an integrator of cognition and emotion (Femenia et al. 2012). Findings from previous studies suggest that alpha-tocopherol may be an exogenous factor regulating adult hippocampal neurogenesis (Ambrogini et al. 2016). In the present study, among the behaviors examined regarding locomotor activity, anxiety level, and learning performance, TBI and PTZ-induced seizures were found to cause an increase in anxiety levels only by increasing the number of closed arm entries in the elevated plus maze test. Alpha-tocopherol administration prevented the increase in anxiety levels associated with TBI and PTZ-induced seizures.

Research has shown that TRPM4 interacts with SUR1 to form a novel ion channel, the SUR1-TRPM4 channel, and that this channel plays a critical role in the pathophysiology of various acute central nervous system injuries (Woo et al. 2013). SUR1-TRPM4 channels promote cytotoxic edema formation. They are also involved in accidental necrotic death triggered by ATP depletion or reactive oxygen species and participate in the formation of ionic and vasogenic edema through blood–brain barrier disruption (Tosun et al. 2013). In a subarachnoid hemorrhage model, endothelial upregulation of TRPM4 was associated with inflammation and BBB permeability (Tosun et al. 2013)and, in a spinal cord injury model, with capillary rupture and secondary hemorrhage (Gerzanich et al. 2009). Based on these data in the literature, it has been stated that TRPM4 may play a critical role in acute CNS injuries with various etiologies (Mehta et al. 2015). In our study, consistent with the literature, alpha-tocopherol administration significantly reduced SUR1 and TRPM4 levels, which increased with TBI + PTZ group This is a finding that alpha-tocopherol may reduce secondary damage caused by TBI + PTZ-induced seizures.

In an experimental study on status epilepticus, 4 days of alpha-tocopherol treatment, initiated immediately after status epilepticus, was found to reduce the expression of the proinflammatory cytokines IL-1β and TNF-α (Ambrogini et al. 2014). Another study by the same team found that high-dose alpha-tocopherol treatment initiated after a seizure in status epilepticus caused a strong reduction in neuroglial activation and neuronal degeneration (Ambrogini et al. 2016). These studies have shown that alpha-tocopherol administered after a seizure can attenuate the neuroinflammatory and neurodegenerative processes that may be caused by status epilepticus. In the present study, the increased levels of inflammatory cytokines (TNF-α, IL-1β, and IL-6) associated with TBI and PTZ-induced seizures were significantly reduced by alpha-tocopherol treatment. This finding, consistent with the literature, indicates that alpha-tocopherol treatment has an anti-inflammatory effect in the TBI and PTZ-induced seizures.

Previous studies suggest that vitamin E, particularly alpha-tocopherol, can be considered an exogenous factor affecting neurogenetic processes in the dentate gyrus of the adult rat hippocampus, possibly leading to changes in gene expression through signal transduction modulation (Ambrogini et al. 2016). The TBI + PTZ group in our study had affected neurons exhibiting degenerative and apoptotic features such as hypereosinophilic cytoplasm, chromatin condensation, and pericellular clearing. Classic apoptotic bodies with fragmented nuclei and cytoplasmic blebs were rare. In the TBI + PTZ + tocopherol group, a limited number of affected cells were seen throughout the cortex, hippocampus, and dentate gyrus. Experimental rodent studies have reported that high vitamin E intake reduces brain atrophy and DNA damage in mothers exposed to ethanol (Shirpoor et al. 2009). Adult and aged rats supplemented with a tocotrienol-rich diet (200 mg/kg/day) for 8 and 3 months, respectively, showed a reduction in the amount and severity of DNA damage compared to age-matched unsupplemented controls (Taridi et al. 2014). In our study, the number of 8-OHdG-positive cells per slice was increased in the TBI + PTZ group in both CA1 and DG regions compared to the control group. Tocopherol treatment confirmed the protective effect by reducing the number of 8-OHdG-positive cells in both regions compared to the TBI + PTZ group. Collectively, these results suggest that TBI and PTZ-induced seizures are associated with significant oxidative DNA damage in hippocampal neurons and glia and that tocopherol treatment reduces this oxidative stress, highlighting its potential neuroprotective role in the context of TBI and PTZ-induced seizures.

miR-23a-3p is known for its effects on apoptosis and inflammation. Li et al. in an article published in 2020, it was reported that miR-23a-3p was downregulated after TBI in rats (Li et al. 2020). A similar result was obtained in our study. miR-324-5p is localized on chromosome 17p. A study by Ferretti et al. reported that miR-324-5p expression decreases in cerebellar neuronal progenitors but increases with differentiation, thus facilitating cell maturation and growth inhibition. Downregulation of miR-324-5p is genetically determined by the deletion of chromosome 17p associated with Medullablastoma (Ferretti et al. 2008). Similar to this study, our study demonstrated that downregulation of miR-324-5p caused disease in the established TBI and PTZ-induced seizures, and that tocopherol treatment increased miR-324-5p expression, demonstrating its therapeutic efficacy. In an article published in 2018 by Tang et al., it was reported that downregulation of miR-324-5p affects Hedgehog signaling activation in multiple myeloma, leading to disease (Tang et al. 2018). Our study also yielded data similar to and supporting this study.

Epileptogenic processes developing after TBI are closely associated with increased oxidative stress and neuroinflammatory responses in the early period (Eastman et al. 2020; Mukherjee et al. 2020). In the presented experimental study, the significant increase in SUR1 and TRPM4 levels, accompanied by an increase in seizure intensity, frequency, and total seizure duration observed in the TBI and seizures induced by subconvulsive PTZ application, suggests that ion homeostasis is disrupted in the post-traumatic period and that epileptogenic network hyper-excitability develops. The fact that alpha-tocopherol administration reduced seizure intensity and duration without altering the PTZ requirement indicates that the effect modulates the pathophysiological microenvironment created after trauma, rather than directly antagonizing the convulsive process. Indeed, alpha-tocopherol treatment suppresses inflammatory cytokine responses in both serum and brain tissue, and significantly improves oxidative stress parameters in brain tissue, indicating that secondary damage mechanisms supporting the epileptogenic process are limited. In particular, the decrease in the expression of the SUR1–TRPM4 channel complex is thought to be associated with the mitigation of neuronal damage due to cytotoxic edema, ionic imbalance, and energy deficiency. Rebalancing thiol-disulfide homeostasis with alpha-tocopherol can be considered an additional mechanism that reduces epileptogenic fragility by contributing to the preservation of cellular redox signalling and repair capacity. Histopathological findings and the significant decrease in 8-OHdG immunoreactivity support the idea that alpha-tocopherol limits widespread oxidative DNA damage and necrotic/degenerative neuronal processes associated with TBI and PTZ-induced seizures. In addition, it is thought that the regulation of miR-23α−3β and miR-324-5β expression may reflect early molecular reprogramming occurring secondary to the effects of alpha-tocopherol on the oxidative and inflammatory microenvironment. This mechanistic framework suggests that the effects of alpha-tocopherol are not limited to a single biological pathway, but rather simultaneously target the reciprocal interactions between oxidative stress, inflammation, and ion channel regulation. In particular, the simultaneous observation of redox balance restoration and partial normalization in miRNA expression profiles through suppression of SUR1–TRPM4 channel activity reveals that post-traumatic epileptogenic remodelling is a multi-level process. In this context, it seems more appropriate to consider alpha-tocopherol as an agent that limits functional and molecular network instability rather than structural damage in epileptogenesis.

When studies in the literature examining the effect of alpha-tocopherol in TBI models are evaluated together, it is seen that this vitamin exhibits a significant neuroprotective effect. Experimental data reveal that α-tocopherol limits secondary damage, particularly by suppressing oxidative stress (reducing lipid peroxidation, protecting antioxidant enzymes) and reducing the inflammatory response (Inci et al. 1998; Ishaq et al. 2013; Rana et al. 2020). In addition, maintaining neurotransmitter balance and achieving motor-cognitive improvement at the behavioral level suggest that it also has positive effects on functional outcomes (Rana et al. 2020). At the mechanistic level, α-tocopherol has been reported to support axonal regeneration, suppress Nogo-A/NgR pathways, and reduce histopathological damage (Yang et al. 2013). Furthermore, it has been shown to protect learning and memory processes by modulating molecular systems associated with synaptic plasticity (BDNF, CREB, CaMKII) (Wu et al. 2010). The findings from the presented study demonstrate that alpha-tocopherol is an early neuroprotective agent in TBI and PTZ-induced seizures through multiple pathways, including oxidative stress, inflammation, and ion channel regulation. Considering all these findings together, it can be stated that alpha-tocopherol has a multifaceted therapeutic potential, supporting improvement at both biochemical and functional levels in seizures that may develop after TBI.

## Limitations

The dose and route of administration of alpha-tocopherol were determined based on previous experimental studies. There is no standard dose regimen for TBI, and dose-dependent effects should be carefully evaluated. Since seizure induction was based on TBI and subconvulsive pentylenetetrazol administration, the independent contributions of trauma and chemoconvulsant exposure cannot be completely separated. Finally, behavioral tests were performed in a fixed order, and potential order effects cannot be ruled out. Although rats are nocturnal animals, the procedures were performed during the daylight period, 09:00–12:00. Although all procedures were standardized across all groups, drug-circadian rhythm interactions were not evaluated in this study.

## Conclusion

In conclusion, the findings of this study demonstrate that alpha-tocopherol is a potential neuroprotective agent contributing to the modulation of early epileptogenic network instability in TBI through multiple pathways, including oxidative stress, inflammation, and ion channel regulation. However, the association between changes in SUR1-TRPM4 expression and miRNA levels and the reduction in seizure severity points to a strong mechanistic relationship rather than a causal link. Therefore, further functional and molecular studies are needed to definitively distinguish whether the observed effects of alpha-tocopherol are directly through these molecular targets or secondary effects resulting from its contribution to the normalization of the oxidative and inflammatory microenvironment.

## Data Availability

All source data for this work (or generated in this study) are available upon reasonable request.
